# An Overview of Orchid Protocorm-Like Bodies: Mass Propagation, Biotechnology, Molecular Aspects, and Breeding

**DOI:** 10.3390/ijms21030985

**Published:** 2020-02-02

**Authors:** Jean Carlos Cardoso, Cesar Augusto Zanello, Jen-Tsung Chen

**Affiliations:** 1 Laboratory of Plant Physiology and Tissue Culture, Department of Biotechnology, Plant and Animal Production, Centro de Ciências Agrárias, Universidade Federal de São Carlos, Rodovia Anhanguera, km 174, CEP 13600-970 Araras, SP, Brazil; jeancardctv@gmail.com; 2 Masterscience degree by Programa de Pós Graduação em Produção Vegetal e Bioprocessos Associados, Centro de Ciências Agrárias, Universidade Federal de São Carlos, CEP 13600-970 Araras, SP, Brazil; cesarzanello1@gmail.com; 3 Department of Life Sciences, National University of Kaohsiung, Kaohsiung 811, Taiwan

**Keywords:** biotechnology, breeding, mass propagation, Orchidaceae, protocorm-like bodies, somaclonal variation, somatic embryogenesis

## Abstract

The process through induction, proliferation and regeneration of protocorm-like bodies (PLBs) is one of the most advantageous methods for mass propagation of orchids which applied to the world floricultural market. In addition, this method has been used as a tool to identify genes of interest associated with the production of PLBs, and also in breeding techniques that use biotechnology to produce new cultivars, such as to obtain transgenic plants. Most of the molecular studies developed have used model plants as species of *Phalaenopsis*, and interestingly, despite similarities to somatic embryogenesis, some molecular differences do not yet allow to characterize that PLB induction is in fact a type of somatic embryogenesis. Despite the importance of species for conservation and collection purposes, the flower market is supported by hybrid cultivars, usually polyploid, which makes more detailed molecular evaluations difficult. Studies on the effect of plant growth regulators on induction, proliferation, and regeneration of PLBs are the most numerous. However, studies of other factors and new technologies affecting PLB production such as the use of temporary immersion bioreactors and the use of lighting-emitting diodes have emerged as new tools for advancing the technique with increasing PLB production efficiency. In addition, recent studies on *Phalaenopsis equestris* genome sequencing have enabled more detailed molecular studies and the molecular characterization of plantlets obtained from this technique currently allow the technique to be evaluated in a more comprehensive way regarding its real applications and main limitations aiming at mass propagation, such as somaclonal variation.

## 1. Introduction

Orchids (Family Orchidaceae) represent one of the two largest plant families, including from 736 [1] to 899 genera and 27,800 accepted species names [2] and over 100,000 hybrids produced by artificial pollination [3]. In addition to their unquestionable botanical and ecological importance, orchids participate in current cultivation systems using high-tech horticulture, grown in environments with good climate control, especially temperature, which allows the induction of flowering regardless of the time of year, especially aiming at the scheduled supply of potted and cut flowers in the competitive world flower market. Some species of orchids, such as the genera *Dendrobium, Gastrodia,* and *Bletilla*, have also been used for medicinal purposes, using the basis of traditional Chinese medicine [4] and some *Vanilla* species is also used for food purposes [5].

In this economic context, family Orchidaceae currently represents one of the most important in the world commercial floriculture, with emphasis on the genus *Phalaenopsis* as well as its interspecific hybrids, which is currently the main potted flower marketed in the main world flower markets. To have an idea of the importance of this genus in the expansion of world floriculture, only in the Dutch market, the largest in the world, in 2014, 121 million pots of *Phalaenopsis* were sold generating approximately US$ 500 million [6]. In addition to *Phalaenopsis*, other genera of economic importance to floriculture include the genera *Cattleya*, *Dendrobium,* and *Oncidium* and their hybrids [7,8,9] as well as *Cymbidium* and *Vanda* used for production of potted or even cut flowers.

Despite the individual importance of these genera, a commercial classification for orchids must be set separately from the botanical classification. This is because although genera have a greater genetic and morphological contribution to commercial plants, most commercial flower production of these genera occurs through the production of hybrids from interspecific crosses, which include the use of crosses between species of the same genus, but also species of different genera (intergeneric hybrids) [9]. An example of this case is the very frequent use of *Doritis* in crossings with *Phalaenopsis*, generating the hybrid genus known as *Doritaenopsis* [10,11]. Nevertheless, commercially these hybrids are all called *Phalaenopsis* because considering the morphological similarity and commercialization value, there is no commercial justification for separation into two classes.

Another justification for the separation of botanical and commercial classification is the recent changes of genera in many species, including those of commercial importance and resulting from the advancement of available molecular techniques that allow genetic rather than just morphological comparisons [1]. An example would be the genera *Laelia* and *Sophronitis*, commonly used in crossings with the genus *Cattleya* to incorporate hybrids with red, yellow and orange flowers, little present in *Cattleya*. Both *Laelia* and *Sophronitis* have undergone more than one change in their names in the last decade, with new changes possibly still remaining due to advances in molecular markers and phylogenetic aspects related to this complex and diverse plant family [12,13].

Thus, it is important to highlight this botanical difference from the commercial one, due to the complexity of the family and its high hybridization capacity. Thus, using as an example the commercial classification encompassing these genera includes not only the genus, but its many hybrids used for the genetic improvement and development of new cultivars for the world floriculture. When mentioning *Cattleya*, this includes genera such as *Laelia*, *Sophronitis*, *Broughtonia*, *Epidendrum*, *Encyclia*, *Caularthron*, among other correlates and with possible hybridization with *Cattleya*. The same occurs in *Oncidium*, in which plants of different genera such as *Brassia*, *Ionopsis*, *Odontoglossum*, *Miltonia*, among others [14] are used for breeding intergeneric hybrids and many commercial hybrids are the result of combinations of more than two genera.

In few plant families it is possible to obtain so many viable and fertile combinations of progenies from very different morphologically species and genera. This allows breeders to incorporate numerous traits of interest into a single plant, which brings the innovative aspect of flower production as well as the advance in breeding, using these same mostly fertile hybrids for the advancement of generations of crosses and obtaining new hybrids. This high hybridization capacity may be a result of the specific process of embryogenic development and later protocorm development that occur in orchids [15]. In other species, it has been reported that lack of hybridization and hybrid seed abortion is associated with disruption of proper endosperm development or mismatch between endosperm development and embryo [16]; and zygotic embryogenesis in family Orchidaceae, embryo development occurs in the absence of endosperm [15].

After obtaining the hybrid of commercial interest, propagation is the factor that defines the time for this hybrid to be available in the market for clonal propagation, which ensures the maintenance of the selected characteristics in propagated plants, quickly, on a large scale and allowing the production of plantlets throughout the year. These propagation characteristics, in addition to ensuring the quality of the plantlets produced, also aim to maintain the commercial scale necessary to meet the target market. The only viable technique that combines all these characteristics has been in vitro micropropagation of orchids [17].

Among the in vitro cultivation techniques used for the in vitro seedling or plantlets production of orchids, it can be used the in vitro asymbiotic germination and micropropagation techniques aiming at the large-scale production of clonal plantlets.

Asymbiotic germination involves the in vitro inoculation and germination of orchid seeds with the aid of a sucrose-containing culture medium [18,19], under conditions free of microorganisms; including those symbionts that assist in germination, especially under natural conditions, a technique known as symbiotic germination, which can be done in vitro [19,20], ex vitro, or in situ and which, unlike asymbiotic, considers the use of symbiotic microorganisms to assist in the germination and early development of newly germinated seedlings, and lacking nutritional reserves to support early seedling development [20,21].

Techniques involving the germination of orchid seeds under in vitro conditions are especially used in: Conservation and production of seedlings of native species; germination of seedlings from crosses aiming at genetic improvement and production of new orchid cultivars [8]; aiming at the production of protocorms in order to study somatic embryogenesis in vitro, also known as protocorm-like bodies or simply PLBs [17,22]. They can also be used for commercial propagation and seedlings production, but with high genetic variability inherent in the family Orchidaceae, including commercial groups used for flower production [8].

In vitro germination of orchids makes it possible to increase the efficiency of conservation and breeding programs, since in vitro germination rates higher than 70% are commonly reported [23], while in ex vitro conditions under natural environmental conditions, these rates hardly exceed 5% germinated seeds [24]. This is especially due to the fact that orchid seeds do not contain nutritional reserves [25], and the embryo and seedlings at early germination are highly dependent on symbiosis with microorganisms known as mycorrhizae, which nutritionally supply these plants during a long time until the complete establishment of the seedling in the natural environment [26]. In *Serapias vomeracea* orchid, in symbiosis with *Tulasnella calospora* there was observed a differential gene expression related to organic nitrogen transport and metabolism, showing the nutritionally supply of fungus to orchids in early development of protocorms [27].

A characteristic of the in vitro asymbiotic germination of orchids is the formation of the so-called protocorms, prior to budding, mainly containing the first leaves and undeveloped stem, followed by the roots [25] and later on with the development of the leaf and pseudobulb.

The term protocorm-like bodies (PLBs) is used as a reference to this type of protocorm-producing germination, characteristic of orchids. The main difference between the germination and the sexual reproduction process, which includes the fertilization process, zygotic embryogenesis, followed by the germination and formation of protocorms, is that PLBs comes from somatic tissues, therefore being considered a type of vegetative propagation.

The production of PLBs, therefore, can be compared to a specific type of somatic embryogenesis that occurs in orchids, and the anatomy, development and characteristics of cells and some cell wall markers at the beginning of PLB formation are similar to those in the development of protocorms in orchids [28]. These authors observed that in non-embryogenic callus of *Phalaenopsis* orchids, the inability to synthesize some cell wall components such as the JIM11 and JIM20 epitopes resulted in loss of morphogenic capacity of these calli, and the correct formation of the cell wall is directly associated with the ability of cell division and elongation in these cell types. In contrast, embryogenic calli synthesized these components, similar to what occurred in zygotic embryogenesis [28].

Despite these anatomical and cellular similarities between PLB induction and zygotic embryogenesis, molecularly, zygotic embryogenesis in *Phalaenopsis aphrodite* is considered different from PLB formation, and that induction of PLBs follows a different route from the embryogenic program [29]. One explanation for these differences is a consequence of the degree of speciation for the development of the embryogenic program in orchids, which follows a very specific pattern and different from the conventional embryogenic program occurring in species of other families, such as the absence of endosperm development and gene expression for establishing symbiotic relationships during seed germination process [15].

Due to these still-present doubts regarding comparisons of zygotic embryogenesis with induction of PLBs in orchids, we have adopted the term IPR–PLB (induction, proliferation, and regeneration of PLBs) as the standard to describe this technique in this paper. IPR–PLBs in orchids have different applications in the world flower industry. Undoubtedly the one with the largest commercial application is aimed at the mass propagation of clonal plants to meet the world’s demanding flower production market, in which orchids play a significant part in both the pot and cut flower market [6,30]. However, other applications such as for species conservation purposes [31] and obtaining transgenic plants [32] can be found in the literature.

Despite a significant amount of studies with IPR-PLB in different orchid species and hybrids, such as *Coelogyne cristata and C. flaccida* [33,34], *Cyrtopodium paludicolum* [35], *Grammatophyllum speciosum* [36] among others, this review has as its main objective to compile the recent studies and advances found in the induction, proliferation and regeneration of PLBs from the two most important genera in the world flower market, especially *Phalaenopsis* and *Oncidium* hybrid groups.

## 2. Genus *Phalaenopsis* and Related

The limited efficiency of clonal multiplication by the induction of shoots from floral stems cultivated in vitro has been one of the main difficulties faced in micropropagation of *Phalaenopsis*, resulting in an increase in the production cost of micropropagated plantlets [37] and associated with falling prices in the international market [6] place in vitro plantlets as the current major cost of producing *Phalaenopsis*. In this sense, the IPR–PLBs can be an important tool in the micropropagation of commercial hybrids of this genus aiming to increase the production efficiency, being necessary to know the main factors involved in each phase of plantlets from PLBs production, e.g., induction, proliferation, and regeneration, which result in efficient clonal and mass propagation techniques for *Phalaenopsis*.

The first studies involving clonal micropropagation of *Phalaenopsis* were conducted by [38,39,40] using *Phalaenopsis amabilis* as a model. Soon after, [41] concluded that leaf segments obtained from inflorescence buds grown in vitro when grown in New Dogashima Medium (NDM) [41] medium supplemented with 0.1 mg L^−1^ NAA (Naphthaleneacetic Acid) and 1.0 mg L^−1^ BA (6-Benzyladenine) could generate up to 10,000 PLBs within a year. Ref. [42] also reported PLB regeneration from a callus induction phase (indirect somatic embryogenesis) using Vacin Went medium [43] supplemented with 20% coconut water and 4% sucrose with the hybrid *Phalaenopsis* Richard Shaffer ‘Santa Cruz’.

In orchids, PLBs are suggested to be somatic embryos due to the morphological similarity and developmental pattern observed between them and the zygotic embryos [42,44]. Besides that, ontogenetic studies based on histological and histochemical methods developed by [28] compared the early developmental pattern of zygotic embryos and PLBs, which led to the conclusion that cytological characteristics and cell wall markers were similar in the early developmental stages of both zygotic embryos and PLBs, which would justify saying that PLBs are somatic embryos. Still, histological analyses made by [45] also showed that the formation of PLBs occurs directly on the epidermal surface of the leaf segment with a cluster of meristem cells in constant division and without connection with the leaf vascular system, which is interesting from a commercial point of view, since it ensures the health of plants obtained through PLBs [46,47,48,49] and enable success of genetic transformation [50,51].

In several plant species, some genes that are involved in somatic embryogenesis, known as *SERK* (somatic embryogenesis receptor-like kinase), are described. Ref. [52] characterized and analyzed the expression of 5 of these genes in *Phalaenopsis* and which were described by the authors as *PhSERK*. According to this study, the expression of these 5 genes was observed in various parts of plants (root, leaf, apical bud, and flower meristem) as well as during seed germination and PLB induction. According to the authors, PLBs segmented and grown in secondary PLB-inducing medium showed high *PhSERK5* expression during the third week, when secondary PLBs became visible, suggesting that this SERK transcription may be closely associated with the acquisition of embryogenic competence during formation of PLBs. It is noteworthy that transformed *Arabidopsis* plants with overexpression of the *AtSERK1* gene showed high capacity for induction of somatic embryos in in vitro culture [53], showing that this gene is indeed involved in somatic embryogenesis, at least in *Arabidopsis*.

Although cytological features indicate that a PLB is a somatic embryo and studies have shown *PhSERK* gene expression during PLB induction [52], transcriptome studies developed by [29] analyzing gene expression in *Phalaenopsis aphrodite* concluded that PLBs are molecularly distinct from zygotic embryos. According to the authors, PLBs share different transcriptomic signatures from zygotic embryos, and early processes of PLB development show a distinct regeneration program, not following the embryogenesis program. In addition, the authors report that the SHOOT MERISTEMLESS gene, a class I KNOTTED-LIKE HOMEOBOX gene, probably plays an important role in PLB regeneration and should be further investigated.

The genetic transformation with *AtRKD4* gene, which encode proteins with RWP-RK transcription factor and is associated to early embryogenic pattern in *Arabidopsis thaliana* [54], also increases the number of PLBs produced in leaves of this *Phalaenopsis* ‘Sogo vivien’ [55] and *Dendrobium phalaenopsis* [56] transgenic plants.

Recent studies with *Phalaenopsis equestris* genome sequencing [57], with 2n = 2x = 38 and 29,431 predicted protein-coding genes and *Phalaenopsis* Brother Spring Dancer ‘KHM190’ [58], 2n = 2x = 38 and 41,153 protein coding genes, make room for further detailed studies on the identification and expression of genes involved in the production of PLBs from different types of somatic tissue in orchids, which can be compared with other model species and in which the embryogenic pathway is already better elucidated, similar to the studies already carried out that brought new discoveries about flowering and the development of floral organs [58].

**Figure 1:**
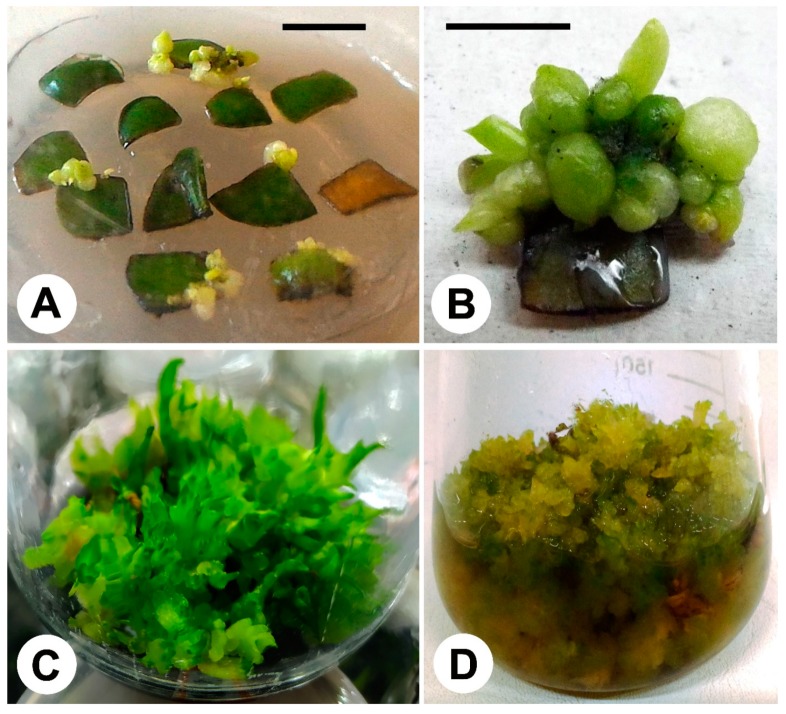
Induction, proliferation and regeneration of protocorm-like bodies in *Dendrobium* and *Phalaenopsis* orchids. Protocorm-like bodies (PLBs)-directly induced from leaf segments of *Phalaenopsis* hybrid ‘501’ **(A)** obtained from young in vitro shoots from inflorescence nodal segments and details of secondary PLBs (**B**) obtained in New Dogashima Medium (NDM) culture medium. Proliferation of PLBs in agar (**C**) and liquid (**D**) MS½ culture medium of *Dendrobium* ‘Hybrid 3’. Bars = 1 cm. Unpublished photos of Cesar A. Zanello (A,B) and Jean C. Cardoso (C,D).

Among the several factors that regulate somatic embryogenesis in *Phalaenopsis*, the absence of light is described as responsible for the PLB induction step [59]. After maintaining the leaf segments for 60 days in the dark, it is possible to observe at the ends of the segments the formation of embryo-like structures, still with a yellowish-white color (Figure 1A). After about 15 days under 14 h light photoperiod, PLBs change color to light green and dark green (Figure 1B) and after 90 days subjected to light there is the onset of differentiation of PLBs with leaf primordia to their complete differentiation with leaf and root formation. The PLBs also could be induced from shoots and proliferate in solid (Figure 1C) or liquid medium under shake agitation (Figure 1D).

From these observations, it is possible to infer that the absence of light plays an important role in the induction of PLBs, just as light influences the differentiation of PLBs into plantlets. Also, according to [60], the type of light used can also optimize the regeneration of PLBs, with the use of red and white LED combined with sucrose as a carbohydrate source, or blue and white LED with trehalose as the carbohydrate source, which had the best response for the regeneration of PLBs. However, only 17.5% of papers described a dark-period to induce PLBs, while 67.5% used light period (12-16-h photoperiod) to induce and regeneration of PLBs in *Phalaenopsis* (Table 1).

Besides the influence of light, another admittedly important factor in the induction of PLBs in *Phalaenopsis* and orchids in general is the genotype [61]. This means that under the same cultivation condition, the induction responses of PLBs may be significantly different [62], which is still considered a limitation of the technique. Ref. [30] evaluated the induction of PLBs in two commercial hybrids (Ph908—red-painted yellow flowers and RP3—dark red) of *Phalaenopsis* and reported significant differences in both percentage of PLB leaf segments (45% and 10%, respectively) as in the number of PLBs per leaf segment (25 and 2 PLBs, respectively).

Regarding the type of explant, leaf segments of plants grown in vitro have been the most suitable for induction of PLBs in *Phalaenopsis* (45% of papers; Table 1), but there are reports of protocols that used in vitro roots of *P.* ‘Join Angle × Sogo Musadian’ cultivated in MS½ medium supplemented with NAA, BAP, and IAA (0.5 ppm, 5 ppm, and 0.5 ppm, respectively) and up to 49.33 PLBs/explant [63].

Segmentation made in leaf segments of *Phalaenopsis* to induce PLBs results in a process called phenolic oxidation, which is the release of polyphenol oxidase (PPO) [103] and other compounds toxic to plant tissue, which may cause its death [74], consequently reducing the induction of PLBs. The immersion of leaf segments in solution of cystine and ascorbic acid during the leaf segmentation stage is reported as a way to reduce the release of these compounds capable of impairing the formation of PLBs [74].

One of the influential factors in the induction of PLBs that has been widely evaluated is the concentrations and possible combinations of plant growth regulators (PGRs). Based on the current literature, successful induction of PLBs seems to be mainly influenced by cytokinin BA (6-benzyladenine) and cytokinin-like compound TDZ (thidiazuron), and in some cases the combination of these cytokinins with an auxin [30,45] also proved beneficial. Protocols citing the use of cytokinin BA recommend concentrations between 0.5 mg L^−1^ [78] and 20 mg L^−1^ [67]. For the induction of PLBs with the use of TDZ, the recommended concentrations range from 0.25 mg L^−1^ [30] to 3.0 mg L^−1^ [72]. With the combined use of cytokinins and auxins, the most commonly used auxin is NAA, which varies in concentration from 0.1 mg L^−1^ [45,74] to 1.0 mg. L^−1^ [30,82]. 

Ref. [104] reviewed the influence of auxins in orchids, including in PLBs and concluded that auxins is important for callus induction and PLB formation and proliferation, while is inhibitory for PLB regeneration into shoots.

As already described, the addition of PGRs is critical to the success of the PLB induction and regeneration technique in *Phalaenopsis*. Cytokinin-like compound such as TDZ (47.5%) and BA (35%) was the most PGRs used to IPR-PLB technique (Table 1). Nevertheless, the use of these regulators may also result in somaclonal variation. This variation can be assessed by morphological, physiological, biochemical traits or molecular markers [105]. Using Random Amplified Polymorphic DNA (RAPD) markers, [82] reported 17% dissimilarity between PLBs and the parent plant in *P. bellina*. Ref. [89] observed 20% dissimilarity after 20 weeks of cultivation in *P. gigantea* using ISSR (Inter Simple Sequence Repeats) markers, leading to the conclusion that PLB proliferation should be done for up to 16 weeks to reduce somaclonal variations and morphological changes. It should be noted that changes in alleles will not always result in phenotypic changes [106], so the variations observed by the markers will not always cause some kind of morphological change in plants. 

According to [107], the combination of red light and far red contributes to decrease endoreduplication rates during PLB induction and regeneration, and consequently may reduce somaclonal variations during mass propagation processes.

Bioreactors could be used to improve the proliferation of PLBs in *Phalaenopsis*. The authors of [108] obtained 18,000 PLBs from 1000 PLBs sections using 0.5 or 2.0 L volume of air per volume of medium min^−1^.

## 3. *Oncidium* Hybrids Group

According to the World Checklist of Selected Plant Families of the Kew Botanical Garden, in December 2019, there are 374 accepted names of *Oncidium* species with more than 90% of accepted names allocated in Southern America and the last in Northern America. In addition to the species, thousands more interspecific and intergeneric hybrids have been registered with the Royal Horticultural Society and are used in the commercial production of cut and pot flowers worldwide [9,109]. Different chemical and physical factors alter the response to PLB induction in *Oncidium*. Using *Oncidium* ’Gower Rampsey’ shoot tips, [109] observed a higher percentage of shoot tips induced to produce PLBs (96.7%) in monochromatic red-light emitting diodes (RR), compared to blue LED (83.3%) and fluorescent white light (76.7%) used as control. However, the use of RR, as well as green LEDs, increased in inhibition of differentiation of PLBs into green buds, while blue LEDs enhanced differentiation. Associated with this response, the authors also observed that in blue light, PLBs contained higher contents of carotenoids, chlorophyll, soluble proteins, lower amounts of soluble sugars and carbohydrates. The authors further argue that in red LEDs, where a higher PLB induction response was obtained, there was a greater accumulation of soluble sugars, starch and carbohydrates, while in blue light, where there was a greater differentiation of PLBs, there was a greater accumulation of proteins and pigments such as chlorophylls and carotenoids.

PGRs are one of the most tested factors in IPR–PLBs in *Oncidium* (Table 2). Benzyladenine (BA) at 2.0 mg L^−1^ + 0.2 mg L^−1^ Naphthaleneacetic Acid (NAA) has been shown to be the most efficient treatment for inducing PLBs in *Oncidium* ’Sweet Sugar’ apical and axillary buds [110] and the combination of 0.1 mg L^−1^ BA + 0.2 mg L^−1^ ANA resulted in better response for *Oncidium* Aloha ’Iwanaga’ [111]. In this context, BA can be used efficiently to obtain PLBs in *Oncidium* in 31.8% of the papers, and auxin NAA is the one most used along with BAP (Table 2).

Interestingly, [112] reported the individual and combined effects of BA and NAA PGRs at different stages of in vitro induction, proliferation and regeneration of PLBs on *Oncidium* sp. These authors identified that previous callus production in culture medium containing 2,4-D at 1.0 mg L^−1^, prior to induction, was beneficial for the production of PLBs from in vitro shoots, and from callus it was possible to observe up to 98 PLBs/callus cluster using 0.75 mg L^−1^ NAA, while only 28.2 PLBs/shoot cluster were directly obtained using the combination of 0.5 + 0.5 mg L^−1^ NAA and BA, respectively. The use of 1.0 mg L^−1^ NAA alone allowed PLB proliferation (up to 79.2 PLBs/sample), while the addition of 1.0 mg L^−1^ BA resulted in shoot bud formation (up to 12.4 shoots/PLB). Similarly, [113] observed that the concentration of 2.0 mg L^−1^ BA resulted in the highest number of shoot buds obtained from PLBs (4.3/PLB) in *Oncidium* ‘Sweet Sugar’.

Thidiazuron (TDZ) also appears to have a pronounced effect on direct induction of PLBs in *Oncidium* leaf segments and were reported in 54.5% of the papers (Table 2), being higher for the percentage of explants directly forming PLBs (60–75%) and number of PLBs per explant (10.3–10.7) compared to other cytokinins such as kinetin, zeatin, 2-isopentenyladenine and BA itself [114]. Ref. [115] reported direct regeneration of PLBs from mainly the epidermis and cut regions of young leaf segments of *Oncidium* ‘Gower Ramsey’ using TDZ alone (0.3–3.0 mg L^−1^), rather than BA in the culture medium, while the combination 2,4-D and TDZ was not beneficial for induction of PLBs. The production of PLBs from tissue damaged regions of inflorescence segments (65%) of *Oncidium* ‘Gower Ramsey’ using 3 mg L^−1^ TDZ [116] has also been reported. A similar experiment using the same cultivar observed that calli from root apexes and stem segments produced PLBs in medium containing 0.3–3.0 mg L^−1^ TDZ, being beneficial the addition of NAA for the formation of embryos n root and leaf calli [117], being a tissue-specific response.

Other PGRs as GA_3_ is reported as an inhibitor of PLB induction in *Oncidium*, while the use of antigibberellins, as ancymidol and Paclobutrazol, increased the percentage of leaf explants with PLBs and the number of PLBs obtained [118].

**Figure 2:**
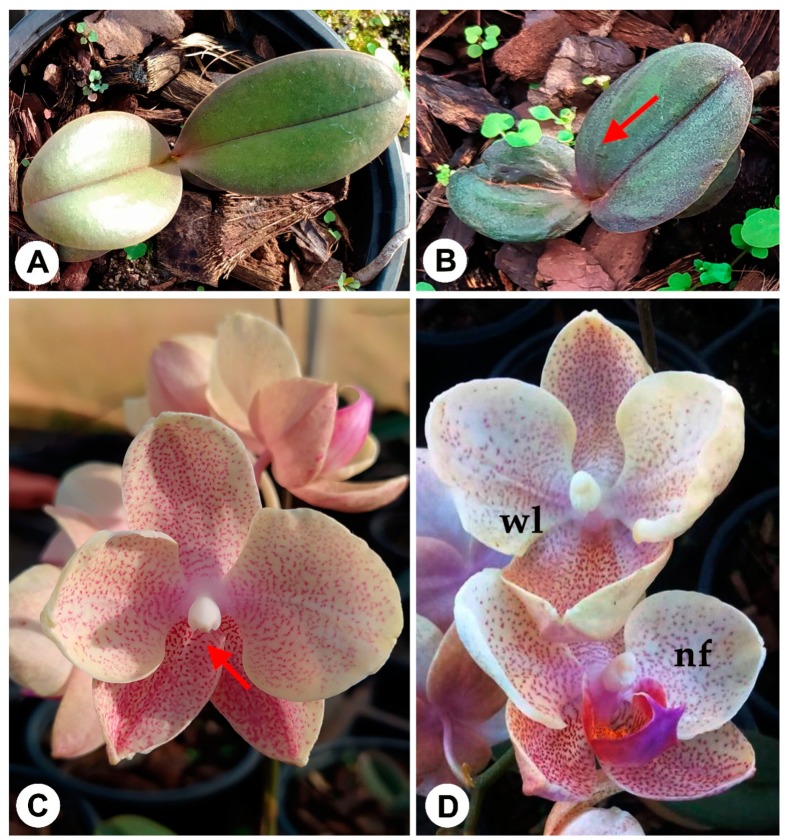
Somaclonal variations observed in *Phalaenopsis* induction, proliferation and regeneration of protocorm-like Bodies in *Phalaenopsis* Hybrid “908”. Normal vegetative developed plant (**A**) and somaclonal variation observed in vegetative development with “creased leaves” (red arrow) (**B**); (**C**,**D**), Normal vegetative developed plants with somaclonal variations in flower development, with first and last flower without of labellum (red arrow, wl) in the same inflorescence with normal flowers (nf). All figures are unpublished photos from J.C.C.

The use of liquid medium, rather than semi-solidified with Agar, is also an alternative for in vitro PLB proliferation (Figure 2). Ref. [113] used 5 L balloon-type air-lift bioreactor to provide mass propagation of *Oncidium* ‘Sweet Sugar’, and show that this system provides 326.3 g PLBs and growth ratio of 10.2, and is more efficient than semi-solid (2.7 g PLBs and Growth ratio of 3.4) and liquid-agitated flask culture (3.5 g PLBs and growth ratio of 4.4). In bioreactor, the lag phase was observed in the first 10-d culture, accompanied by a sharp drop in pH (5.7 to 4.7) and EC (3.2 to 1.5 mS cm^−1^) in the first 20-d of cultivation, followed by an intense mass growth from 10 to 40 days of cultivation, when the pH increased again to 5.9. An interesting fact was the dynamics of sugars in the culture medium, and a fast and drastic reduction of sucrose in the medium was observed, from 27 (day zero) to 5.5 (day five), 1.2 (day 10) and zero (day 20), associated with a substantial increase in glucose and fructose in the first 10 days of cultivation, with the exhaustion of these sugars at 40 days of cultivation, when the PLBs entered the stationary phase, demonstrating that during a certain period the PLBs release invertases in the culture medium to reduce sugars, and these are metabolized during the exponential phase of production of PLBs [113].

Another study conducted in a gelled medium by [124] observed that the use of 2% fructose resulted in 95% explants containing PLBs in *Oncidium* Gower Ramsey or 2% glucose resulted in 85% explants containing PLBs in *Oncidium* Sweet Sugar [124]. However, for the number of PLBs per explant, the best results were obtained with 2–3% sucrose (31.1–33.7 PLBs/explants), demonstrating that sucrose is the most suitable sugar for IPR–PLB. The use of other types of sugars, cellobiose, maltose and trehalose do not result in benefits for number of PLBs from callus in *Oncidium* Gower Ramsey [122] or for direct production of PLBs from young leaves [124].

There are no doubt about the application of PLBs in mass clonal production of *Oncidium* [132], but recent studies also showed and confirmed the presence of somaclonal variation in *Oncidium* obtained from IPR–PLBs [133], similar to observed with *Phalaenopsis* genus.

## 4. Some News with *Cymbidium*, *Dendrobium,* and Others

The most of results obtained with *Phalaenopsis* and *Oncidium* were similar to reported with other species of orchids of importance in floriculture, as *Cymbidium* and *Dendrobium* genera, such as the main PGRs used for IPR–PLBs. As example, the combination of cytokinin BA (5.0 mg L^−1^) and auxin NAA (2.5 mg L^−1^) were used to induce PLBs (20.55 PLB per primary protocorm) in *Cymbidium mastersii* protocorms [134]. Thin cell layers (TCL) from different types of tissues was a technique used to improve the production of PLBs in *Cymbidium* [135], *Dendrobium* [136,137], *Oncidium* [129], and *Phalaenopsis* [93].

In *Dendrobium*, a wide and complete study about molecular research was exhaustively carried out by [138], and considered especially the identification, classification and breeding of *Dendrobium*. Similarly, other study with micropropagation of *Dendrobium* was realized by [17] and concluded that PLBs were used as explants in 21.8% of studies, and together with nodal or nodal segments explants is one of the major method used for *Dendrobium* micropropagation.

Thidiazuron was also an important PGR for induction of PLBs in *Dendrobium* orchids, but the response to different cytokinins depends on genotype. In *Dendrobium aqueum*, only the cytokinin 2iP [*N*-6-(2-isopentyl) adenine] at 1.5 mg L^−1^ proved it efficiency in production of PLBs (42.7 PLBs per explants) from callus, compared to other cytokinins BA, Kin and Zea, and cytokinin-like compound TDZ. These authors also observed that arginine at 25 mg L^−1^ increased direct somatic embryogenesis, instead of callus derived PLBs [137]. Meta-Topolins, a natural aromatic type of cytokinin, were also reported used in induction and regeneration of PLBs in *D. nobile*, which combined with 0.5 mg L^−1^ NAA resulted in best PLBs formation (92%) and shoots/explants (9.2) [139]. These same authors observed that addition of polyamines, such as spermidine and putrescine increased regeneration of shoots from PLBs and secondary PLB formation. 

In our laboratory, PLBs of *Dendrobium* Hybrid ‘H3’, could be induced and proliferated in one-step, and obtained from in vitro shoots, using liquid MS½ medium with 1.0 mg L^−1^ BA, and under agitation of 80 rpm (Figure 1D). 

## 5. Applications of IPR–PLB Technique on Orchid Propagation and Breeding and Main Limitations of the Technique

Induction, proliferation, and regeneration of PLBs in orchids have many advantages to conventional micropropagation by shoot proliferation or use of shoots from inflorescence stalk segments as in *Phalaenopsis* [140], as increased rate of proliferation/multiplication [141] and single-cell derived PLBs [123], which could be used for propagation, but also for breeding purposes and to obtain disease free plantlets. 

In breeding programs using in vitro techniques, PLBs could be used to obtain autotetraploid plants with use of anti-mytotic agents as oryzalin [142] and colchicine [143], and to obtain mutants by the use of chemical mutagens as sodium azide [144] or physical mutagens as gamma-irradiation [145]. 

PLBs can be also used for transformation protocols and successful protocols were developed and obtained stable transgenics with target characteristics for floriculture [146,147]. In genetic transformation of orchids, the use of PLBs derived directly from individual epidermal cells resulted in solid transgenic plants with clonal identity of *Oncidium* Sharry Baby ‘OM8’ [32], an exceptional advantage over PLBs from callus and with multicellular origin [126], which may result in the emergence of somaclonal variants [42] and chimeric tissues when used for genetic transformation, which are difficult to characterize and separate [32]. Using this technique, these authors reported 33–43% PLBs expressing the β-glucuronidase gene (GUS) and obtained six lineages that amplified the transgenes pepper ferredoxin-like protein (pflp) and hygromycin phosphotransferase (hpt) using the particle bombardment technique. *Agrobacterium tumefasciens*-mediated transformation has also been successfully used in the production of transgenic plants of *Oncidium* ‘Sharry Baby OM8’ and *Oncidium* Gower Ramsey using the induction of secondary PLBs from in vitro-maintained PLBs [148,149].

From a phytosanitary point of view, it is known that the use of seeds for in vitro asymbiotic sowing of orchids is a real way to obtain virus-free seedlings in orchids from contaminated mother plants, as observed for *Cymbidium* species [150,151]. Ref. [152] confirmed on a large scale (1000 plants) that in vitro plants from seeds are free of *Cymbidium Mosaic Virus* (CyMV) and *Ondontoglossum Ringspot Virus* (ORSV).

The technique of culturing apical meristems may also be effective in eliminating viral diseases in orchids, but it requires great manual skill for excision of tiny meristems leading to contamination-free tissue [153]. These requirements and the individual characteristics of viral diseases may lead to breakthroughs in the technique, which may result in in vitro plantlets containing viral diseases, as reported in *Brassolaeliocattleya*, *Cattleya*, *Dendrobium*, *Epicattleya*, *Oncidium,* and *Mokara* grown in vitro, for which CyMV virus was reported to be present in 27.6% of 880 plantlets evaluated, while ORSV was not detected in these samples [152].

Furthermore, in genera such as *Phalaenopsis*, the most commercially important in the world, only stem apex culture may not be effective in completely eliminating important viral diseases in the crop [140], and may still result in the need to kill the mother plant to obtain the apical meristem, since these plants are monopodial and have poorly developed stem [150]. In this sense, in vitro IPR–PLBs is an alternative to the production of virus-free clonal plants in orchids. In *Phalaenopsis* hybrid ‘V3’, Ref. [140] obtained PLBs from stem apexes of donor plants contaminated with *Ondontoglossum* ringspot virus and *Cymbidium* mosaic virus, and observed that the first PLBs produced directly from the stem apex had 31.25% PLBs with viruses, identified by the enzyme-linked immunosorbent assay (ELISA) and RT-PCR and were only eliminated in the process after some subcultures. The PLBs identified as virus-free were subcultured in PLB proliferation medium, and in the second subculture 18.18% positive PLBs were identified for both viruses. Only in the third subculture of PLB proliferation, it was possible to obtain 100% virus-free PLBs, which remained until the end of the experiment.

PLBs can also be used for orchid propagation using the synthetic seed technique and for cryopreservation. In *Dendrobium* ‘Sonia’, the use of PLBs stored at 4 °C for 15 days in the pro-meristematic and leaf primordium stages and encapsulated with 3–4% sodium alginate + 75–100 mM CaCl_2_*2H_2_O resulted in 100% germinated PLBs, with the appearance of the first leaf at 22–27 days and the first root at 30–35.8 days, and the technique can be replicated with similar results for *Oncidium* ‘Gower Ramsay’ and *Cattleya leopoldii* [154]. 

In *Dendrobium candidum* and *Dendrobium nobile*, PLBs have also been used to increase the production of bioactive compounds. In *D. nobile*, an increase was observed in the production of secondary metabolites such as phenols, flavonoids and alkaloids extracted from PLB-micropropagated plants, when compared to the mother plant [139]. In *D. candidum*, the increase in methyl-jasmonate elicitor concentrations, although resulting in a proportional reduction in PLBs mass gain, increased the concentrations of alkaloids, polysaccharides, phenols and flavonoids when used between 75 and 100 µM [155].

Although the IPR–PLB technique is widely used for large scale plantlet production, breeding and conservation, some difficulties still limit the wider use of the technique on a commercial scale. Among the main limitations are the high genotype-dependence of PLB induction and proliferation responses in vitro, and the occurrence of undesirable somaclonal variations, which greatly hinder the proliferation of clonal propagation of PLBs for a wide range of commercial cultivars available and required by the market.

Ref. [30] used NDM culture medium plus TDZ (0.25 mg L^−1^) and NAA (1.0 mg L^−1^) and observed distinct responses between ’908’ genotype (45% explants with PLBs and up to 25 PLBs/leaf segment) and ’RP3’ genotype (10% explants with PLBs and only 2 PLBs/leaf segment), the latter being highly recalcitrant to the induction and proliferation of PLBs from leaf segments of plants grown in vitro. A study by [59] also noted important differences between the PLBs induction responses between *P. amabilis* (up to 50% explants with PLBs and 15.6 PLBs/explant) and the commercial cultivar *P. nebula* (80% explants with PLBs and up to 5.3 PLBs/explant). The same occurred in another study with the same cultivars, in which the cytokinin types and concentrations that resulted in the highest percentage of explants with PLBs were 13.32 µM BAP in *P. amabilis* (80%) and 13.62 µM TDZ in *P. nebula* (65%). The largest number of PLBs per explant was obtained with 13.62 µM TDZ in *P. amabilis* (7.8 PLBs/explant) and 4.65 µM Kin in *P. nebula* (16 PLBs/explant) [77].

Ref. [156] point out that one of the biggest difficulties in *Phalaenopsis* micropropagation by PLBs is that not all genotypes respond to a single protocol and the same cultivation conditions, and often result in plants with undesirable characteristics. Ref. [41] compared eight cultivars of *Phalaenopsis* and *Doritaenopsis* to obtain PLBs from shoot tips of inflorescence stalk buds with best percentage of PLB formation in four genotypes using 1.0 mg L^−1^ BAP (26.9–71.4% depending on genotype), while two respond better with 2.0 mg L^−1^ (60–75% explants with PLBs) and one produced 50% PLBs independently of the concentration of BAP (1, 2, or 5.0 mg L^−1^). Testing other four genotypes authors reported ranges from 7.1% to 40% of PLBs formation only in NDM culture medium, while in ½MS only two cultivars produced PLBs [41].

Ref. [156] have been associated undesirable characteristics observed in some plantlets with the identification of somaclonal variants from PLBs, which can be morphologically identified even at the shoot bud regeneration and in vitro plantlet production stage. According to [157], the occurrence of SV in the IPR–PLBs technique is higher than that observed from adventitious bud propagation, and that most commercial laboratories use a maximum of three generations of PLBs subcultures to avoid high frequencies of somaclonal variations in this type of propagation.

In our laboratory conditions, using leaf segments from in vitro plantlets to obtain PLBs (Figure 1A,B) somaclonal variations are observed in rooting phase of PLB-derived plantlets of *Phalaenopsis* ‘Ph908’, while were not observed in plantlets derived from shoot-proliferation using inflorescence stem nodal segments (Figure 2A). The main symptoms were the limited development of plantlets that remains in acclimatized plantlets, with morphological abnormalities in leaves (Figure 2B), also observed and called as ‘creased leaves’ by [66] and flowers deformities as absence of lip in some flowers of the inflorescence (Figure 2C,D) possibly associated with mutations rather than epigenetic variations.

Ref. [139] used induction of PLBs from pseudostems from in vitro germinated *Dendrobium nobile* plants in MS + 1.5 mg L^−1^ TDZ and 0.25% activated charcoal medium and verified 94% explants producing PLBs and up to 11.6 PLBs/explant. These authors observed a somaclonal variation rate close to 6% in the obtained plants, being the main cause of the somaclonal variations detected by molecular markers Random amplified polymorphic DNA (RAPD) and Start codon targeted (SCoT), attributed by the authors to the use and exposure time to TDZ.

Although the cytokinin-like compound TDZ is appointed as one of the major causes of SV in orchid PLB induction, there were some contradictory reports. 

As example, the cytokinin Kinetin at 1.5 mg L^−1^ resulted in increases of somaclonal variations frequency of PLBs in *Dendrobium* Sabin Blue, detected by ISSR and DAMD molecular markers, when compared with use of TDZ at 4.0 mg L^−1^ added activated charcoal [158].

In addition, [159] observed somaclonal variants in *Phalaenopsis* True Lady ‘B79-19’, obtained from the induction of PLBs and from young leaves obtained from in vitro plants in VW culture medium containing only BA and NAA as phytoregulators, i.e., without using TDZ. These authors also reported that variant plants were discarded during in vitro subcultures (not quantified), and out of the plants obtained and without morphological variations in the leaves, only 20 out of a total of 1360 obtained (1.5%) were somaclonal variants, indicated by the different flowers of the original clone. 

Also the use of topolins *meta*-Topolins (*m*T) and *meta*-Topolins Riboside (*m*TR), a natural aromatic cytokinin reported as reducing phytotoxic effects in micropropagation, it use not solved the problem of somaclonal variation obtained in vitro [160] and, although was reported increasing efficiency of PLB induction it use not resulted in absence of somaclonal variation in orchids [139].

These observations with other cytokinins PGRs diminish the importance of TDZ as the unique or main factor for VS inducing in orchid IPR–PLBs, and include other causes, such as the differential susceptibility of genotype and the number of subcultures under proliferation stage of PLB production. 

Genotype susceptibility is appointed one of the main factors lead to VS in *Phalaenopsis* and *Doritaenopsis* orchids micropropagation, ranging from zero to 100% SV depending on genotype and is not exclusive of the PLB technique [72,161]. Similarly, [70] also observed that some genotypes of *Phalaenopsis* not presented any variants, while others showed until 47.9% of variants. Among them, most of SV in this genus were reported in flowering stage [161], by modification of inflorescence and flower characteristics, such as the perloric and semi-perloric mutants observed in *Phalaenopsis* Zuma Pixie ‘#1’, *P.* Little Mary and *Doritaenopsis* Minho Diamond ‘F607’ [162]. Lose of part of flowers were also reported, such as pollinia [162] and absence of labellum (Figure 2C,D). 

Ref. [161] evaluated until the flowering stage (1.0–1.5 years after acclimatization) plants of 10 genotypes of *Phalaenopsis* and *Doritaenopsis* hybrids micropropagated by the PLB technique, and subcultured in vitro for 5 to 10x and identified the presence of seven types of VS, possible to be identified only at the flowering stage. The plants had deficiencies or divergences in the petals and sepals or in the development of the inflorescence, but with similar vegetative development in relation to the mother plant. These authors observed that the produced VS were not polyploid mutants, maintaining the same amount of genetic material as the mother plants.

Although most of SV was reported in flowering stage, transcript analysis by Real-Time PCR demonstrated that mutants has also many other alterations in factors of transcription and transcripts were detailed reported in *Phalaenopsis* and *Doritaenopsis* by [162]. In *Oncidium* ‘Milliongolds’ were also observed chlorophyll SV (whole yellow or with streaked leaves) in vegetative development of in vitro plantlets [133]. 

Another factor related to the origin of VS in PLBs in orchids is the phase in which VS occurs. It has been reported that in the proliferation phase, undesirable VS induction from PLBs occurs at a higher intensity and frequency, and it is necessary to establish a number of subcultures to keep the VS frequencies low in clonal propagation. Ref. [92] reported increases in SV after the third subcultures of PLBs in proliferation medium (NDM + 0.1 mg L^−1^ TDZ and 10 mg L^−1^ chitosan) with same ISSR profile until third subculture, 95% at fourth and 80% at fifth subculture of PLBs. 

The use of RAPD molecular markers (total of 1116 bands) did not allow the identification of these somaclonal variants in these plants, but isozyme pattern analysis demonstrates the difficulty of observing mutations in materials obtained from PLBs using RAPD molecular markers and the occurrence of conclusion errors or even underestimated data of somaclonal variants in the confirmation of clonal origin in other studies conducted with these markers [159].

Ref. [82] also used RAPD markers to analyze the clonal origin of PLBs and induced seedlings in in vitro leaf segments of *Phalaenopsis bellina* in ½MS medium with 3.0 mg L^−1^ TDZ. They observed that most somaclonal variants are obtained at the proliferation/multiplication phase, with no VS observed in the origin phase of the PLBs of the mother plant.

Analyses of SCoT and Target Region Amplification Polymorphism (TRAP) markers also showed the presence of somaclonal variants in *Dendrobium* Bobby Messina PLBs cryopreserved or not [163].

These differences in the frequencies of VS observed in different orchid species and genotypes are probably associated with higher sensitivity of different genotypes to the occurrence of mutations. Ref. [164] observed that the frequency of VS at the vegetative and reproductive stages in *Phalaenopsis* PLBs was dependent on the genotype used. These authors observed that there was a reduction in DNA methyltransferase (Dnmt)-related gene expression in *Phalaenopsis* ‘Little Mary’ VS.

Current advances in molecular marker techniques allow increasing the number of tools and the accuracy of these analyses and the greater possibility of identifying possible VS. There is little information about wide molecular genome characterization in *Oncidium*, and [133] used specific-locus amplified fragment sequencing (SLAF-seq) to analyze possible variations in single-nucleotide polymorphisms (SNPs) in *Oncidium* ‘Milliongolds’ obtained by PLBs grown for 10 years and observed high rates of variation and that adjacent SNPs adenine and thymine were more frequent than those related to guanine and cytosine, with prominence of mononucleotideInDels. 

Ref. [157] isolated two most expressed transposable elements and identified a new Instability Factor (PIF)-like, one of which, called PePIF1 was identified by similarity to the *Phalaenopsis equestris* genome sequence, and which was transposed in the somaclonal variants of cultivars of *Phalaenopsis* from micropropagation, which resulted in the insertion of new genes identified and sequenced by the authors.

## 6. Conclusions

Induction, proliferation, and regeneration of PLBs (IPR–PLBs) in orchids is one of the most promising techniques to replace current conventional micropropagation techniques, in particular because it has wide application in clonal conservation, propagation, breeding, and phytossanitary-cleaning of elite plants used in the flower market. Although many authors used somatic embryogenesis to describe IPR–PLBs technique or their origin, recent molecular studies about the origin route of PLBs, at least in *Phalaenopsis* orchids, showed that IPR–PLBs routes are not the same of somatic embryonic origin. Some limitations of IPR–PLBs in orchids such as low repeatability of responses due to high genotype dependence and the presence of somaclonal variations (SV) still limit their large-scale use in the production of clone plantlets. Although the main causes of SV described in papers were the genotype-sensibility, the use of cytokinin thidiazuron and subsequent PLBs proliferation, only genotype sensibility looks conclusive, because SV was also observed in protocols using other cytokinins, such as BA and Kin. Nevertheless, the new findings associated with the identified instability factors, associated with the recent sequencing of the *Phalaenopsis equestris* genome, and the use of new molecular tools that increase the accuracy of quantitative identification analyses and the causes of somaclonal variation, are in agreement with the evolution of this technique, which represents the tool of greatest potential today to replace other less efficient micropropagation techniques in the production of plantlets in orchids.

## Figures and Tables

**Table 1:** Compliance of studies with induction, proliferation and regeneration of PLBs (IPR-PLBs) with *Phalaenopsis* and *Doritaenopsis*.

Species or Hybrids	Origin and Age of Explants	Culture Media	Growth Conditions	Main Results	Evaluation and Detection of SV	Reference
12 cultivars of *Phalaenopsis*	Shoot tips derived from flower stalk buds	NDM added 10 g L^−1^ sucrose, 2 g L^−1^ Gelrite, 0.1 mg L^−1^ NAA and 1–5 mg ^L−1^ BA	23 ± 1 °C, 14-h photoperiod, 33 µmol m^−2^ s^−1^	93–100% survival rate of explants, 33–40% PLB formation, green color of PLBs showed multiplication, 27–28% PLBs formed shoots	Non-evaluated	[41]
*Phalaenopsis* Nebula	Calluses derived from 1–2 months protocorms	MS½ + 100 mg L^−1^ myo-inositol + 0.5 mg L^−1^ niacin and pyridoxine + 0.1 mg L^−1^ thiamine + 2.0 mg L^−1^ glycine + 170 mg L^−1^ NaH_2_PO_4_ + 20 g L^−1^ sucrose + 2.2 g L^−1^ Gelrite, pH 5.2	26 ± 2 °C, 16-h photoperiod, PPFD 28–36 µmol m^−2^ s^−1^	Both TDZ and BA were able to induce PLBs in calluses, but interestingly equal number of PLBs per callus (74) was obtained when callus was transferred to free-PGR medium	Not observed any phenotypic abnormality and no chromosome number alterations were observed in 2–3 months plantlets	[64]
*Phalaenopsis* Hybrid with pink striped flowers	Section transversely cutted from apical meristems (2-mm in size) of PLBs obtained from leaf segments	Liquid Hyponex modified medium (Kano, 1965—1 g L^−1^ of 6.5N − 4.5P − 19K + 1 g L^−1^ 20N − 20P − 20K + 1% potato homogenate)	25 ± 2 °C, 16-h photoperiod, PPFD 60 µmol m^−2^ s^−1^, white fluorescent light, under shaker at 100 rpm or temporary our continuous immersion bioreactor system	100 ml medium per 0.5 g inoculum under agitation (9.2 PLBs/PLB section) or air-lift balloon with 10.0 g inoculum (12.6 PLBs/PLB section); charcoal filter attached to bioreactor increased to 17 PLBs/PLB section; Hyponex medium increased percentage of PLB regeneration, rooting and fresh weight of plantlets	Non-evaluated	[65]
9 genotypes of *Phalaenopsis*	Shoot tips from flower stalk buds and callus from cell suspension cultures	Shoot tips to PLBS, NDM + 2 g L^−1^ Gellan gum, pH 5.4; Cell suspension, liquid NDM + 58.4 mM sucrose; Induction of PLBs from calluses, NDM + 29.2 μM sucrose + 2 g L^−1^ Gellan gum	23 ± 1 °C, 14-h photoperiod, 33 µmol m^−2^ s^−1^, cell suspension culture were obtained in liquid medium under agitation 0f 80 rpm	44.4% PLB formation from shoot tips were obtained with 0.5 µM NAA and 4.44 µM BA and 29.2 mM sucrose; increases in sucrose concentration (58.4 mM increased callus formation); calluses could induced to PLBs with 29.2 mM sucrose	The type and frequency of morphological variants were large dependent on genotype: in *P*. Snow Parade and *P*. Little Steve any variants was reported, while 47.9% variants were observed in *P.* Reichentea	[66]
*Phalaenopsis* Tinny Sunshine ‘Annie’; ‘Taisuco Hatarot’; Teipei Gold ‘Golden Star’; Tinny Galaxy ‘Annie’	Young leaf segments (10 × 5 mm) derived in vitro shoots from flower stalk nodes	induction of PLBs: MS½ + 10% coconut water/Proliferation of PLBs: different saline formulation + 2 g L^−1^ peptone + 3% potato homogenate + 0.05% activated charcoal + 30 g L^−1^ sucrose	Temp 25 ± 1 °C, 16-h photoperiod by cool white fluorescent lamps, PPFD 30 µmol m^−2^ s^−1^; liquid media in shaker at 50 rpm	70–90% of explants with PLBs depending on cultivar; 85% explants with PLBs and 12 PLBs/explant with 88.8 µM BA + 5.4 µM NAA; 45 g L^−1^ sucrose showed highest number PLBs per explant (6) and low light intensity (10 µmol m^−2^ s^−1^) resulted in best PLBs induction (90%) and number of PLBs/explant (12); liquid with cotton raft support Hyponex medium increased PLBs proliferation (20.5 PLBs)	Non-evaluated	[67]
*Doritaenopsis* ’New Candy’ × (*D.* ’Mary Anes’ × *D.* ’Ever Spring’	Leaf segments 1 mm thick from three months old leaves from in vitro plantlets	MS½ + 20% coconut water + 10 mg L^−1^ adenine sulphate + 2.3 g L^−1^ Gelrite, pH 5.5	1 week in dark at 27 °C followed by 25 ± 1 °C, 16-h photoperiod by cool white fluorescent lamps, PPFD 10 µmol m^−2^ s^−1^	9.0 µM TDZ resulted in best PLB formation (72.3%); Thin leaf segments—1 mm— resulted in best PLB formation (>50%) than thick leaf sections—5 mm (10%) and are correlated with ethylene content (ppm)	Irregular shaped bodies (CLBs) increased with increases in concentrations of TDZ (0.57% at free-PGR to 11.56% at 22.5 µM) and BA (32.14% at 4.4 µM); However, no phenotypic variations were observed in vegetative growth in greenhouse	[68]
*Doritaenopsis* ’New Candy’ × (*D.* ’Mary Anes’ × *D*. ’Ever Spring’	Root tips (<0.5 cm) from 3-months old in vitro plantlets	MS + 20% coconut water + 10 mg L^−1^ adenine sulphate + 2.3 g L^−1^ Gelrite, pH 5.5	Temp 25 °C, cool white fluorescent lamps, PPFD 30 µmol m^−2^ s^−1^, 16-h photoperiod	TDZ at 2.3 µM showed best PLB formation (47.2% of root tips with 2–6 PLBs each) compared to BA and Zea; most of PLBs originated from cortex tissues of root	Non-evaluated	[69]
*Phalaenopsis* Snow Parade and Wedding Promenade, *Doritaenopsis* New Toyohashi	Cell suspension from calluses	NDM + 2 g L^−1^ gellan gum, pH 5.4	23 ± 1 °C, 14-h photoperiod, 33 µmol m^−2^ s^−1^, cell suspension culture were obtained in liquid medium under agitation 0f 80 rpm	The response were genotype-dependent: Glucose at 58.4 mM and sucrose at 29.2 mM showed several increases in number (>2000) and fresh weight of PLBs for P. Snow Parade, while glucose at 14.6–29.2 mM showed highest number of PLBs in *P.* Wedding Promenade	Non-evaluated	[70]
*Phalaenopsis* ’Little Steve’	Leaf explants (1cm2) derived from flower stalk buds eighteen-month-old in vitro plants	MS½ added 4.54 μM TDZ, 100 mg L^−1^ myo-inositol + 0.5 mg L^−1^ niacin + 0.5 mg L^−1^ pyridoxine + 0.1 mg L^−1^ thiamine + 2.0 mg L^−1^ glycine + 1000 mg L^−1^ peptone + 2.2 g L^−1^ Gelrite + 20 g L^−1^ sucrose, pH 5.2	Dark for 2 months followed by 16-h photoperiod	40% explants with PLBs; not reported the number of PLBs per explant	Non-evaluated	[71]
*Phalaenopsis amabilis* var. formosa	Leaf tip segments obtained from in vitro germinated seedlings and leaf-derived nodular masses	M½ S added 3 mg L ^−1^ TDZ, 100 mg L^−1^ myo-inositol + 0.5 mg L^−1^ niacin + 0.5 mg L^−1^ pyridoxine + 0.1 mg L^−1^ thiamine + 2.0 mg L^−1^ glycine + 1000 mg L^−1^ peptone + 2.2 g L^−1^ Gelrite + 20 g L^−1^ sucrose, pH 5.2	Temp 26 ± 1 °C; 16-h photoperiod	93.8% explants with PLBs and 19.4 PLBs per explant for leaf tip segments; 5.4 proliferation rate and 13.8 PLBs per explant for leaf-derived embryogenic masses	Non-evaluated	[72]
*Phalaenopsis gigantea*	Trimmer base protocorms 1 mm from 150-d in vitro germinated protocorms	XER medium (Ernst, 1994) + 20 g L^−1^ fructose + 1% agar, pH 5.7	Temp 25 ± 2 °C, under continuous illumination from cool fluorescent lamps, PPFD 20–50 µmol m^−2^ s^−1^	Trimmed protocorms increased PLBs proliferation (56.8%) and number of PLBs/protocorm (4.24) using 15% coconut water and 2.5 g L^−1^ activated charcoal, compared to untrimmed (4.56% and 0.56 PLB/protocorm) and shoot regeneration from PLBs were increased using only coconut water at 10% (33.56% shoot formation)		[73]
Alba flower hybrid’ of *Phalaenopsis*	Nodular masses	NDM culture medium added 1.0 mg L^−1^ BA and 0.1 mg L^−1^ NAA, 100 mg L^−1^ myo-inositol + 1.0 mg L^−1^ (niacin, pyridoxine, thiamine, cysteine, calcium pantothenate) + 0.1 0 mg L^−1^ biotin + 20 g L^−1^ sucrose + 2.0 g L^−1^ Phytagel, pH 5.8	Not reported growth conditions	8.5 PLBs per explant; not reported percentage of explants with PLBs	Non-evaluated	[74]
*Phalaenopsis amabilis* cv. ’Cool Breeze’	Inflorescence axis thin sections	MS½ added 2,0 mg L^−1^ BA, 0,5 mg L^−1^ NAA, 2% sucrose, 10% coconut water, 2 g L^−1^ peptone and 1 g L^−1^ activated charcoal		20 PLBs/explant after 12 weeks	Non-evaluated	[75]
*Phalaenopsis* var. Hawaiian Clouds × *Phalaenopsis* Carmela’s Dream	Clumps of callus (8 mm diameter)	NDM culture medium added 1 mg L^−1^ TDZ, 10 g L^−1^ maltose, 2.8 g L^−1^ Gelrite	Temp 25 ± 2 °C, in the dark	52.5% callus with PLBs	Non-evaluated	[76]
*Phal. amabilis; Phal.* ’Nebula’	Cut end of leaf explants (1.0 cm length); clonal plantlets of *P. amabilis* and in vitro germinated seedlings for *P.* ’Nebula’	MS½ added 3 mg L^−1^ TDZ, 100 mg L^−1^ myo-inositol + 0.5 mg L^−1^ niacin + 0.5 mg L^−1^ pyridoxine + 0.1 mg L^−1^ thiamine + 2.0 mg L^−1^ glycine + 1000 mg L^−1^ peptone + 2.2 g L^−1^ Gelrite + 20 g L^−1^ sucrose, pH 5.2	Temp 26 ± 1 °C; dark for 60-d (induction) 45-d for subculture period;	50% explants with PLBS and 8.2 PLBs/explant for *P. amabilis*; 80% explants with PLBs and 3.5 PLBs for *P.* ’Nebula’	Non-evaluated	[59,77,78]
10 genotypes of *Phalaenopsis*	Basal portion of sectioned horizontally protocorms (3–5 mm) were placed upward in contact with the culture medium	3.5 g L^−1^ HyponexTM #1 + 1 g L^−1^ tryptone + 0.1 g L^−1^ citric acid + 1 g L^−1^ activated charcoal + 20 g L^−1^ sucrose + 20 g L^−1^ homogenized potato + 25 g L^−1^ homogenized banana + 7.5 g L^−1^ agar, pH 5.5	Temp 25 ± 2 °C, 16-h photoperiod with PPFD 10 µmol m^−2^ s^−1^	22% of sectioned protocorms induced PLBs and 17.5 PLBs per responsive protocorms were obtained	High endopolyploidy were observed in *Phalaenopsis* protocorms; from 22 diploid protocorms used as explant, 34.1% of derived-PLBs were polyploidy at first cycle and 51.7% at second cycle of proliferation	[79]
*Phalaenopsis violacea*	Leaf segments (1 × 1 cm) from in vitro shoots derived from flower stalks	MS½ + 5% banana extract	Temp 25 °C, 16-h photoperiod, PPFD 40 µmol m^−2^ s^−1^ by white fluorescent tubes	70% of leaf segments formed PLBs with 0.8 µM BAP, while TDZ were able to induce PLBs only in 40% of explants and BAP (0.6 µM) was more effective to PLBs proliferation than TDZ and Zea	Non-evaluated	[80]
*Phal. amabilis* cv. Lovely (purple flowers)	Young emerging leaves from in vivo plants	MS1/2 + 2% sucrose + 10% coconut water + 2 g L^-1^ peptone + 1 g L^-1^ activated charcoal + 2.2 g L^-1^ Gelrite, pH 5.6;	Temp 24 ± 1 °C, cool white fluorescent light, PPFD 30 µmol m^−2^ s^−1^, 16-h photoperiod	2.0 mg L^−1^ BA and 0.5 mg L^−1^ NAA resulted in 75.5% explants formed PLBs and 10 PLBs/explant; MS½ + 10% coconut water + 150 mg L^−1^ glutamine showed best proliferation rate of PLBs (200.5 PLBs/explant)	Non-evaluated	[81]
*Phalaenopsis bellina*	*In vivo* leaf	MS½ + 100 mg L^−1^ myo-inositol + 0.5 mg L^−1^ niacin and pyridoxine + 0.1 mg L^−1^ thiamine + 2.0 mg L^−1^ glycine + 3.0 mg L^−1^ TDZ + 2% sucrose + 3.0 g L^−1^ Gelrite + 10% fresh banana extract, pH 5.6	PLBs from leaf (S0), PLBs proliferation after 3 months (S1) and after six months (S2)	Efficiency of induction and regeneration of PLBs not presented by authors	Minimal dissimilarity in *P. bellina* by RAPD markers; S0 presented 96% similarity, S1 87% and S2 80% similarity to the mother plant	[82]
*Phalaenopsis bellina*	Young leaves (1.5 cm^2^) of a nursery plant	MS½ + 2% sucrose + 100 mg L^−1^ myo-inositol + 0.5 mg L^−1^ niacin and pyridoxine + 0.1 mg L^−1^ thiamine + 2.0 mg L^−1^ glycine + 10% fresh ripen banana extract + 3.0 mg L^−1^ TDZ + 3.0 g L^−1^ Gelrite, pH 5.6	Temp 25 ± 2 °C, 14-h photoperiod for 12–16 weeks	71.9–78.1% explants with PLBs; 14.3–14.8 PLBs per flask; MS1/2 was the best for PLB proliferation compared to VW	Non-evaluated	[83]
*Phalaenopsis gigantea*	Leaf tip segments (1.0 cm length) from in vitro germinated seedlings	NDM culture medium, sucrose 20 g L^−1^ + 1.0 mg L^−1^ NAA and 0.1 mg L^−1^ TDZ	Temp 25 ± 2 °C, 12-h photoperiod for 6 weeks	The authors only report that NAA and TDZ treatment was the best for callus induction and PLBs after 6 weeks of culture.	Non-evaluated	[84]
*Phalaenopsis amabilis* cv. ’Golden Horizon’	Young emerging leaves from in vivo plants	MS½ + 2% sucrose + 10% coconut water + 2 g L^−1^ peptone + 1 g L^−1^ activated charcoal + 2.2 g L^−1^ Gelrite, pH 5.6	Temp 24 ± 1 °C, cool white fluorescent light, PPFD 30 µmol m^−2^ s^−1^, 16-h photoperiod	BA at 2.0 mg L^−1^ combined with NAA 0.5 mg L^−1^ resulted in 80.5% explants with PLBs and 15 PLBs/explant; MS½ + 10% coconut water + 150 mg L^−1^ glutamine showed best proliferation rate of PLBs (250.5 PLBs/explant)	Non-evaluated	[85]
*Phalaenopsis* Gallant Beau ’George Vasquez’	Longitudinally bisected PLBs (2–3 mm in diameter) and 2-months old	Miracle Pack® culture system with liquid VW + 20% coconut water without sucrose, pH 5.3	Temp 25 °C, 16-h photoperiod, PPFD 45 µmol m^−2^ s^−1^, plant growth fluorescent lamps, under magnetic fields	Although higher Fresh weight of PLBs was obtained with 0.1 Tesla–South (237.4 g), best number of PLBs was obtained in control without magnetic fields; 0.15 Tesla for 7 weeks (South) also increased PLB fresh weight, control treatment not differed from the best results using magnetic fields	Non-evaluated	[86]
*Phalaenopsis cornu-cervi*	Leaf segments from in vitro germinated seedlings with 2-months	MS½ added 0.1 mg L^−1^ NAA, 0.1 mg L^−1^ TDZ and 15% coconut water	Temp 25 ± 1 °C; 16-h photoperiod for 45 days	100% explants with PLBs; 35 PLBs per explant	Non-evaluated	[87]
*Phalaenopsis gigantea*	PLBs obtained from leaf tip segments (1.5 cm length) from young leaves	Liquid medium with 20% coconut water, pH 5.4.	Temp 25 ± 2 °C, under 16-h photoperiod using fluorescent lighting 30 µmol m^−2^ s^−1^, 60 rpm rotary shaker	VW medium with 10 mg L^−1^ chitosan resulted in higher number of PLBs (177) and fresh weight of PLBs (8.4 g)	ISSR, non-detected somaclonal variations in *P. gigantea* related to mother plants	[88]
*Phalaenopsis* ’R11 × R10’	Leaves, root tips and stem explants from eight months (plantlets or seedlings?)	MS½ + 15% coconut water + 0.01% activated charcoal + 0.03% polyvinylpyrrolidone (PVP) + 88.8 µM BA + 5.37 µM NAA + 0.025% Phytagel, pH 5.6–5.8	Temp. 25 °C, 16-h photoperiod	Stem segments were interesting explant for PLB induction; sucrose at 3% (71.2 PLBs) was more effective than maltose (39 PLBs) in PLBs proliferation	Non-evaluated	[89]
*Phalaenopsis* Tropican Lady	Young etiolated shoots leaves segments (5 × 10 mm) from flower stalk nodes for induction and PLBs for proliferation	PLB induction: ¼ macroelements and full-strength microelements, glycine and vitamins of MS + 30 g L^−1^ sucrose + 0.5 mg L^−1^ TDZ + 7 g L^−1^ agar / PLB Proliferation: 3 g L^−1^ Hyponex (7-6-19) + 1 g L^−1^ tryptone + 50 g L^−1^ potato homogenate + 50 g L^−1^ banana homogenate + 30 g L^−1^ sucrose + 2 g L^−1^ activated charcoal + 7.5 g L^−1^ agar, pH 5.6	Temp 25 ± 2 °C, under 12-h photoperiod by cool white fluorescent lamps, PPFD 23.2 µmol m^−2^ s^−1^,	Basal part of sectioned of bi or trisectioned PLBs resulted in highest explants with PLB formation (46.8–96.3%) and number of PLBs/explant (15.4–22.9); wounding stimulate ethylene production and gene expression for stimulation of cell division	Non-evaluated	[90]
*Phalaenopsis cornu-cervi*	Whole leaves and leaf-segments (proximal, middle and distal regions) from 120-d old seedlings	MS½ + 3% sucrose + 15% coconut water + 0.23% Gelrite, pH 5.6	Temp 25 ± 1 °C, under 16-h photoperiod, cool white fluorescent lamps, PPFD 20 µmol m^−2^ s^−1^ or pre-treated with 1 week in the dark before photoperiod	Highest percentage of explants with PLBs (30%) and number of PLBs per leaf segment (5.3) were obtained with 9 µM of TDZ under without dark period. Dark period reduced number of PLBs/explant	Non-evaluated	[91]
*Phalaenopsis gigantea*	Leaf tip segments from young leaves of in vitro seedlings	NDM medium added 0.1 mg L^−1^ TDZ, 10 mg L^−1^ chitosan, 0.2% Gelrite and pH 5.7	Temp 25 ± 2 °C, 16-h photoperiod, 33 µmol m^−2^ s^−1^	353 PLBs per explant and 4.8 g PLBs fresh weight	ISSR, SV detected after the subculture four (5 to 20%)	[92]
*Phalaenopsis* hybrids	Intact and transversally divided protocorms (two or four divisions) 1.0–1.5 mm width	MS + 15% coconut water + 7.0 g L^−1^ agar	Temp 25 ± 2 °C, 16-h photoperiod, 25 µmol m^−2^ s^−1^	No PLBs formed in intact protocorms; Middle and Basal part of sectioned protocorms showed 40 and 44% PLB formation and 11.7 and 13.3 PLBs per explant in Free-PGR culture medium, respectively; Four division of protocorms increased PLBs formation and number of PLBs	Non-evaluated	[93]
*P. aphrodite* subsp. *formosana*	in vitro germinated seedlings with 2-months	Using 2-step method: Liquid MS½ for 2 months and then transferred to solid MS (half strength) with 1 cm of medium Liquid MS (half strength) for a further 2 months. All media with 1 mg L^−1^ TDZ.	Temp 25 ± 2 °C; followed by 16-h photoperiod	44 PLBs per seedling	Non-evaluated	[94]
*Phalaenopsis amabilis* cv. ’Surabaya’	Leaf segments from in vitro shoots obtained from inflorescence stalk segments	-	Temp 25 ± 1 °C; 16-h photoperiod, subcultured each 14-d	5 mg L^−1^ BA + 2 mg L^−1^ NAA produced 8.7 number of PLBs and TDZ at 3.0 mg L^−1^ showed 22.45 PLBs	non reported by authors that acclimatized and cultivated regenerated plantlets until flowering stage	[95]
*Phalaenopsis ’*Fmk02010’	Single PLBs	MS with 412.5 mg L^−1^ NH_4_NO_3_ and 950 mg L^−1^ of KNO_3_ + 20;0 g L^−1^ sucrose + 2.0 g L^−1^ Phytagel, pH 5.5–5.8	-	Hyaluronic acid 9 and 12, at 0.1 mg L^−1^, increased percentage of explants with PLBs (100%), PLB number (18.2 to 23.3) and fresh weight of PLBs (0.291 to 0.596 g) compared to control (86.7%, 12.9 and 0.198 g)	no malformation was observed in regenerated plantlets	[96]
*P.* ‘Join Angle × Sogo Musadian’	*In vitro* roots	MS½ added NAA (0.5 ppm), BA (5 ppm) and IAA (0.5 ppm)	Temp 26 ± 1 °C; dark for 1 month (induction) followed by 16-h photoperiod (4 weeks)	49.33 PLBs per explant; not reported percentage of explants with PLBs	Non-evaluated	[63]
*Phalaenopsis* Classic Spoted Pink	leaf segments (1.0 cm^2^) with 90-d obtained from in vitro shoots	MS½ added NAA (0,537μM) and TDZ (13,621μM)	Temp 25 ± 2 °C, dark for 90-d (induction) followed by 16-h photoperiod	The percentage of explants in regeneration and the number of PLBs/explant were not described	Non-evaluated	[45]
*Phalaenopsis amabilis* var. ’Manila’	Leaf segments (1 cm × 0.5 cm) obtained from in vitro flower stalk nodes	MS added 15 mg L^−1^ BA and 3 mg L^−1^ NAA	Temp 25 ± 1 °C; 16-h photoperiod	50.65 PLBs per explant after 6 weeks	Non-evaluated	[97]
*Phalaenopsis amabilis*	Protocorms (4 weeks-old), roots, leaves and stems (6-month-old) cut transversely	NP (New Phalaenopsis) medium added 3 mg L^−1^ TDZ	25 ± 1 ºC with 1000 lux intensity of continuous light; 8 weeks	Protocorm: 100% explants with PLBs and 23.3 PLBs/explant; Leaf: 100% explants with PLBs and 7.75 PLBs/explant; Root: 80% explants with PLBs and 8.25 PLBs/explant; Stem: 100% explants with PLBs and 28.25 PLBs/explant	Non-evaluated	[98]
*Phalaenopsis* ’Fmk02010’	Single PLBs	MS with 412.5 mg L^−1^ NH_4_NO_3_ and 950 mg L^−1^ of KNO_3_ + 2.2 g L^−1^ Phytagel, pH 5.5–5.8	Temp 25 ± 2 °C, 16-h photoperiod, PPFD 54 µmol m^−2^ s^−1^	Highest number of PLBs (54.13) were obtained with Red-White LEDs and with sucrose at 20 g L^−1^ and highest fresh weight of PLBs (0.167 g) was obtained with Red-Blue-White LEDs and trehalose (20 g L^−1^)	Non-evaluated	[60]
*Phalaenopsis* ’RP3’ and ’908’	Leaf segments (0.4–0.5 cm^2^) obtained from in vitro shoots	NDM culture medium added 0.25 mg L^−1^ TDZ (908) or 1.0 mg L^−1^ NAA, 20.0 mg L^−1^ BA and 0.125 mg L^−1^ TDZ (RP3)	Temp 25 ± 2 °C, dark for 60-d (induction) followed by 14-h photoperiod	45% (908) and 10% (RP3) explants with PLBs; 25 and 2 PLBs/explant respectively	Non-evaluated	[30]

NDM: New Dogashima Medium [41]; MS: Murashige and Skoog Medium [99]; Hyponex medium: [100]; XER medium: [101]; VW: Vacin Went medium [43]; NP: New *Phalaenopsis* medium [102]. 2,4-D, 2-4-Dichlorofenoxiacetic acid; BA, 6-Benzyladenine; IAA, 3-Indoleacetic acid; IBA, Indole-3-butyric acid; NAA, Naphtaleneacetic acid; PPFD: Photosynthetically Photon Flux Density; Temp, Temperature; TDZ, Thidiazuron.

**Table 2:** Compliance of studies with induction, proliferation and regeneration of PLBs (IPR-PLBs) technique used with *Oncidium* species and hybrids.

Species or Hybrids	Origin and Age of Explants	Culture Media	Growth Conditions	Main Results	Evaluation and Detection of SV	Reference
*Oncidium varicosum*	Root tips 1.5 mm long from seedlings	Modified VW (replace Fe_2_(C_4_H_4_O_6_)_3_ by 27.8 mg L^−1^ Fe-EDTA + 15% coconut water (PLBs proliferation from PLB) + 1.25 mg L^−1^ NAA (callus and PLB induction), pH 5.5	25 ± 1 °C, Gro-lux bulbs with 16-h photoperiod and 500 lux	Only one callus formed PLB and proliferation of PLBs occurred only in liquid medium with 15% coconut water	Non-evaluated	[119]
*Oncidium* ‘Gower Ramsey’	Leaf segments 5 mm in length from in vitro plantlets leaves of 2–4 cm and 5–7 cm	MS½ + 100 mg L^−1^ inositol + niacin and pyridoxine (0.5 mg L^-1^) + thiamine (0.1 mg L^-1^) + glycine (2.0 mg L^-1^), peptone (1000 mg L^-1^), NaH_2_PO_4_ (170 mg L^-1^), sucrose (20,000 mg L^-1^) + Gelrite (2,500 mg L^-1^), pH 5.2	Temp 26 ± 2 °C, PPFD 28–36 µmol m^−2^ s^−1^, daylight fluorescent tubes, 16-h photoperiod	Donor leaves with 5–7 cm long showed higher percentage formed PLBs (25–35%) and number of PLBs/leaf segment (17–24.4) than 2–4 cm donor leaves (15–25% and 5.3–13.0) using 1–3 mg L^−1^ TDZ; proliferation of PLBs was highest with 0.3 mg L^−1^ TDZ, and regeneration of PLBs showed best response in absence of PGRs	Non-evaluated	[115]
*Oncidium* ‘Gower Ramsey’	Leaves 2–4 and 5–7 cm, stem internodes 5mm and root tips 1 cm	MS½ + 100 mg L^−1^ inositol + niacin and pyridoxine (0,5 mg L^-1^) + thiamine (0.1 mg L^-1^)+ glycine (2.0 mg L^-1^), peptone (1000 mg L^-1^), NaH_2_PO_4_ (170 mg L^-1^), sucrose (20,000 mg L^-1^) + Gelrite (2200 mg L^-1^), pH 5.2: callus phase, 3.0 mg L^−1^ 2,4-D + 3.0 mg L^−1^ TDZ; PLBs, 0.1 NAA + 3.0 mg L^−1^ TDZ	Temp 26 ± 1 °C, PPFD 28–36 µmol m^−2^ s^−1^ white cool fluorescente, 16-h photoperiod	10% and 25% callusing from stem and root tips, 3.38 and 3.86 callus proliferation rate from stem and root tips, until 93.8 callus forming embryos and 29.1 embryos/callus from roots	Different callus lines showed large differential response to PLBs induction (0% to 93.8%) and number of PLBs/explant (0 to 29.1)	[117]
*Oncidium* ‘Gower Ramsey’ and *O.* ‘Sweet Sugar’	Internodes 5 mm length from 15–20 cm inflorescence length	MS½ + 100 mg L^−1^ inositol + niacin and pyridoxine (0,5 mg L^-1^) + thiamine (0.1 mg L^-1^)+ glycine (2.0 mg L^-1^), peptone (1000 mg L^-1^), NaH_2_PO_4_ (170 mg L^-1^), sucrose (20,000 mg L^-1^) + Gelrite (2200 mg L^-1^), pH 5.2	Temp 26 ± 1 °C, PPFD 28–36 µmol m^−2^ s^−1^, daylight fluorescent tubes, 16-h photoperiod	TDZ 1–3 mg L^−1^ increased explants produced PLBs directly in *O.* Sweet Sugar, but not in *O.* Gower Ramsey. Callus from explants on NAA + TDZ both at 1.0 mg L^−1^ showed 19 PLBs/callus. PLBs regeneration into shoots occurred in free-PGR MS½	Non-evaluated	[116]
*Oncidium* ‘Gower Ramsey’	Leaf explants 1 cm in length from two-month old donor in vitro plantlets	MS½ + 100 mg L^−1^ inositol + niacin and pyridoxine (0,5 mg L^-1^) + thiamine (0.1 mg L^-1^)+ glycine (2.0 mg L^-1^), peptone (1000 mg L^-1^), NaH_2_PO_4_ (170 mg L^-1^), sucrose (20,000 mg L^-1^) + Gelrite (2200 mg L^-1^), pH 5.2	Temp 26 ± 1 °C, PPFD 28–36 µmol m^−2^ s^−1^, daylight fluorescent tubes, 16-h photoperiod	Auxins IAA, NAA, IBA and 2,4-D inhibited direct PLB induction, while cytokinins promoted; TDZ 0.3–3.0 mg L^−1^ increased percentage of explants formed PLBs (60–75% in leaf tips and 25–40% in adaxial surfaces, with 9.5–10.7 PLBs/explant	Non-evaluated	[114]
*Oncidium bifolium*	Leaf segments 4 × 4 mm from germinated seedlings	MS½ + 2% sucrose + 2 g L^−1^ Phytagel + 1.0 mg L^−1^ TDZ, pH 5.5	27 ± 2 °C, 14-h photoperiod	25.5% of leaf segments formed PLBs and 12 PLBs/explant	Non-evaluated	[120]
*Oncidium* ‘Gower Ramsey’	Leaf explants 1-cm length from two month old in vitro donor plantlets	MS½ + 1.0 mg L^−1^ TDZ, pH 5.2	Temp 26 ± 1 °C, PPFD 28–36 µmol m^−2^ s^−1^, daylight fluorescent tubes, 16-h photoperiod	Leaf tips and leaves with adaxial surface in contact with culture medium was the best region for PLB induction, sucrose at 10–20 g L^−1^, NaH_2_PO_4_ 170 mg L^−1^, peptone 1.0 g L^−1^ (65–80% explants with PLBs and 10.7 to 11.2 PLBs/explant);	Non-evaluated	[121]
*Oncidium* ‘Gower Ramsey’	Leaf tips 1-cm length from two month old in vitro donor plantlets	MS½ + 100 mg L^−1^ inositol + niacin and pyridoxine (0,5 mg L^-1^) + thiamine (0.1 mg L^-1^)+ glycine (2.0 mg L^-1^), peptone (1000 mg L^-1^), NaH_2_PO_4_ (170 mg L^-1^), sucrose (20,000 mg L^-1^) + Gelrite (2200 mg L^-1^), pH 5.2	Temp 26 ± 1 °C, PPFD 28–36 µmol m^−2^ s^−1^, daylight fluorescent tubes, 16-h photoperiod	GA_3_ inhibited PLB formation, while anti-gibberellins Ancymidol (2.5 mg L^−1^) and paclobutrazol (10 mg L^−1^) increased explants formed PLBs (80–87.5% leaf tips formed PLBs and 154.8–193.2 PLBs/petri dish)	Non-evaluated	[118]
*Oncidium taka*	Axillary buds 0.5–1.0 cm lenght	MS + 3% sucrose + 0.7% agar, pH 5.7–5.8. PLBs induction at 1.0 mg L^−1^ BA + 0.5 mg L^−1^ NAA; PLBs regeneration at 2.0 mg L^−1^ BA + 1.0 mg L^−1^ BA	26 ± 2 °C, 12-h photoperiod, 3000 lux cool white fluorescent light	90% explants with PLBs and 9.4 shoots per culture	Non-evaluated	[122]
*Oncidium* ‘Gower Ramsey’	Shoot tips 2–3 mm length for callus induction and 9-months age callus line for PLBs induction	MS½ + thiamine (1.0 mg L^-1^) + nicotinic acid and pyridoxine (0.5 mg L^-1^) + glycine (2.0 mg L^-1^) + inositol (100 mg L^-1^) + 2% sucrose + 7.5 g L^−1^ Agar, pH 5.7: callus proliferation, 1.0 mg L^−1^ 2,4-D + 0.5–1.0 mg L^−1^ TDZ / PLBs induction, 0.1 mg L^−1^ NAA and 0.4 mg L^−1^ BA with sucrose, maltose or trehalose	callus induction and proliferation in dark for 60-d (induction), subcultured every 2-weeks; PLBs induction, Temp 26 ± 2 °C, PPFD 57 µmol m^−2^ s^−1^, 16-h photoperiod	680–732 g callus FW (1.0 mg L^−1^ 2,4-D and 0.5–1.0 mg L^−1^ TDZ); 1478 PLBs/0.25 g callus (Sucrose 10–20 g L^−1^); 24 to 52.9 efficiency of plantlet conversion from PLBs (trehalose at 20 g L^−1^)	Non-evaluated	[123]
*Oncidium* ‘Gower Ramsey’ and *O.* ‘Sweet Sugar’	Leave tips 1-cm long from in vitro plantlets	MS½ + 1.0 mg L^−1^ TDZ	Temp 26 ± 1 °C, PPFD 28–36 µmol m^−2^ s^−1^ daylight fluorescent tubes, 16-h photoperiod	Leaf tips and Adaxial region of leaves showed most response to PLB formation; 95% explants with PLBs with 20 g L^−1^ fructose in two cultivars; 31.1 (*O.* Sweet Sugar) to 33.7 (*O.* Gower Ramsey) PLBs/explant with 20 and 30 g L^−1^ sucrose, respectively	non evaluated	[124]
Cut flower varieties of *Oncidium*	New lateral buds	MS + 25 g L^−1^ sucrose + 10% coconut water + 7.5 g L^−1^ agar + 3.0 mg L^−1^ BA + 0.3 mg L^−1^ NAA, pH 5.6	25 ± 2 °C, 10–12-h photoperiod, 2000–2500 lux cool white fluorescent light	proliferation of 2.96	Non-evaluated	[125]
*Oncidium* ‘Gower Ramsey’	PLBs from callus	Method described by ref. [123] using 10 g L^−1^ maltose	Callus at 26 ± 2 °C in the darkness; PLBs from callus in 50 µmol m^−2^ s^−1^ for 16-h photoperiod, under blue (455 nm), red (660 nm) and Far-red (730 nm)	2986 PLBs under fluorescent lamps statistically equal to red + blue + far red LEDs (3114 PLBs)	Non-evaluated	[126]
*Oncidium flexuosum*	Leaf apices 0.5 cm in length from 4-m seedlings	MS½ + 30 g L^−1^ sucrose + myo-inositol 100 mg L^−1^ + 5 g L^−1^ agar + nicotinic acid and Pyridoxine (0.5 mg L^-1^) + thiamine (0.1 mg L^-1^) + glycine (2.0 mg L^-1^), pH 5.8	Temp 25 ± 2 °C, PPFD 40 µmol m^−2^ s^−1^, 16-h photoperiod	Darkness for 90-d before photoperiod increased explants regenerating PLBs from 5 (Light) to 80% (Dark) and 10.8 PLBs per explant using 1.5 mg L^−1^ TDZ. Until 29.3 PLBs/explant 60-d after transfer PLBs to free-PGR MS	Non-evaluated	[127]
*Oncidium* ‘Sugar Sweet’	Shoot tips 0.5 mm length for callus induction and PLBs obtained from callus	Callus: MS½ +2.0 mg L^−1^ BA + 0.3 mg L^−1^ NAA + 30 g L^−1^ sucrose + 7.0 g L^−1^ agar, pH 5.7; PLBs proliferation in MS + 30 g L^−1^ sucrose + 1.0 mg L^−1^ BA + 0.2 mg L^−1^ NAA, pH 5.8; PLBs regeneration, MS + 2.0 mg L^−1^ BA + 0.1 mg L^−1^ NAA + 30 g L^−1^ sucrose + 7.0 g L^−1^ agar	PLBs proliferation: 25 °C, 16-h photoperiod, white fluorescent light at 30 µmol m^−2^ s^−1^ at 5 l balloon type air lift bioreactor, 20 g fresh weight PLBs per bioreactor	3335.5 g fresh weight PLBs per vessel and 16.8 growth ratio; until 4.3 shoots/PLB and 1.17 g fresh weight per explant	Non-evaluated	[113]
*Oncidium* ‘Gower Ramsey’	Shoot tips 5 mm for PLB induction and PLBs sections 3–4 mm diameter	PLBs induction: MS½ + 30 g L^−1^ sucrose + 6.0 g L^−1^ agar + 1.0 mg L^−1^ BA / PLBs proliferation: MS + 30 g L^−1^ sucrose + 6.0 g L^−1^ agar + 1.0 mg L^−1^ BA + 0.5 mg L^−1^ NAA	Temp 25 ± 2 °C, 16-h photoperiod	Red LEDs (660 nm) resulted in best induction rate (83.3% explants), Fresh weight (≡ 20 g) and propagation rate (>6) of PLBs, while Blue LEDs showed 90% of differentiation rate of PLBs into shoots	Non-evaluated	[109]
*Oncidium* ‘Gower Ramsey’	Root tips segments 1 cm in length from 6-months old in vitro plantlets	Callus induction: MS½, pH 5.2 / PLB induction: MS½ + 0.1 mg L^−1^ NAA + 3.0 mg L^−1^ TDZ	Temp 25 ± 1 °C, darkness	Age of callus from 0.5 to 2 years resulted in best percentage (80–100%) of callus produced PLBs and number of PLBs/callus (6.2–6.6); the increase in age of callus reduced it embryogenesis capacity	Different callus lines showed large differential response to PLBs induction. However, 3-years old plantlets greenhouse cultivated showed same color, size and morphology of O. Gower Ramsey	[128]
*Oncidium forbesii* (*Brasilidium forbesii*)	Transverse and lateral Thin cell layers 1mm thickness from in vitro germinated protocorms	WPM + 3% sucrose + 0.6% agar, pH 5.8	Temp 25 ± 1 °C/19 ± 1 °C (day/night), 16-h photoperiod, white fluorescent tubes 40 µmol m^−2^ s^−1^	Lateral thin cell layers in culture medium with BA at 2.0 µM increased PLB induction in 64 to 82% explants and both from lateral and transversal TCL at 1.0 µM promoted the number of PLBs obtained/explant (17.1–24.6)	Non-evaluated	[129]
*Oncidium* ‘Gower Ramsey’	PLBs sections obtained from nodal explants from inflorescences	MS½ (full strength MS vitamins) + 1 g L^−1^ tryptone + 20 g L^−1^ sucrose + 1 g L^−1^ activated charcoal + 65 g L^−1^ potato tuber + 8 g L^−1^ agar + 5 μM TDZ (TDZ, vitamins and glycine were filter sterilized)	Temp 22 ± 2 °C, 16-h photoperiod	PLBs regeneration from PLBs section increased with addition of chloro or methyl or nitro derivatives (compounds 5a–5c) using 2–5 µM, from 41 (control) until 95 plantlets per culture bottle using 5 µM of 5c compound	Non-evaluated	[130]
*Oncidium* sp. (Vu Nu Orchids)	In vitro shoots	MS½ + 20 g L^−1^ sucrose + 10% coconut water + agar, pH 5.8	Temp 26 ± 2 °C, PPFD 22.2 µmol m^−2^ s^−1^, 12-h photoperiod	NAA 0.75 mg L^−1^ produced highest number of PLBs/callus (98) and 1 mg L^−1^ BA promoted PLBs regeneration into shoots (12.42/PLB)	Non-evaluated	[112]
*Tolumnia* Snow Fairy	Leaf segments from different in vitro plantlets height and leaf positions	MS½ (with Fe-NaEDTA, vitamins and glycine at full-strength MS) + 100 mg L^−1^ myo-inositol + NaH_2_PO_4_ (170 mg L^−1^), 30 g L^−1^ sucrose + 8.0 g L^−1^ agar, pH 5.2	Temp 25 ± 2 °C, 8-weeks in dark and transferred to dim light, PPFD 5 µmol m^−2^ s^−1^, cool white fluorescent tubes, 12-h photoperiod	Leaves from 1–2 cm plantlet height showed highest explants induced PLBs using 2.0 mg L^−1^ BA (16.7%), but highest number of embryos was obtained with 4.0 mg L^−1^ BA and from plantlets with 2–3 cm (41 PLBs/explant), upper wounding region of bigger PLBs improved PLBs proliferation and number of PLBs per explant	Plants were transferred to plastic pots and flowered after one-year without reports of somaclonal variations in vegetative and reproductive phase	[31]

MS: Murashige and Skoog Medium [99]; VW: Vacin Went medium [43]; WPM: Wood Plant Medium [131]. 2,4-D, 2-4-Dichlorofenoxiacetic acid; BA, 6-Benzyladenine; IAA, 3-Indoleacetic acid; IBA, Indole-3-butyric acid; NAA, Naphtaleneacetic acid; PPFD: Photosynthetically Photon Flux Density; Temp, Temperature; TDZ, Thidiazuron.

## References

[1] Chase M.W., Cameron K.M., Freudestein J.V., Pridgeon A.M., Salazar G., Van den Berg C., Schuiteman A. (2015). An update classification of Orchidaceae. Bot. J. Linn. Soc..

[2] (2019). The Plant List. http://www.theplantlist.org/1.1/browse/A/Orchidaceae/#statistics.

[3] RHS 2019 The International Orchid Register. https://apps.rhs.org.uk/horticulturaldatabase/orchidregister/orchidregister.asp.

[4] Bulpitt C.J., Li Y., Bulpitt P.F., Wang J. (2007). The use of orchids in Chinese medicine. J. R. Soc. Med..

[5] Zuraida A.R., Izzati K.H.F.L., Nazreena O.A., Zaliha W.S.W., Radziah C.M.Z.C., Zamri Z., Sreeramanan S. (2013). A simple and efficient protocol for the mass propagation of *Vanilla planifolia*. Am. J. Plant Sci..

[6] Chen C. (2018). The Fundamental Issue in the Phalaenopsis Industry. http://amebse.nchu.edu.tw/orchids_cultivation21.htm.

[7] Cardoso J.C. (2012). *Dendrobium* ‘Brazilian Fire 101’-New option of color of flowers for the orchid market. Hortic. Bras..

[8] Cardoso J.C., Martinelli A.P., Teixeira da Silva J.A. (2016). A novel approach for the selection of Cattleya hybrids for precocious and season-independent flowering. Euphytica.

[9] Cardoso J.C. (2017). *Ionocidium* ‘Cerrado101’: Intergeneric orchid hybrid with high quality of blooming. Ornam. Hortic..

[10] Ho T.-T., Kwon A.-R., Yoon Y.-J., Paek K.-Y., Park S.-Y. (2016). Endoreduplication level affects flower size and development by increasing cell size in *Phalaenopsis* and *Doritaenopsis*. Acta Physiol. Plant.

[11] Lakshman C., Pathak P., Rao A.N., Rajeevan P.K. (2014). Commercial Orchids.

[12] Van den Berg C. (2014). Reaching a compromise between conflicting nuclear and plastid phylogenetic trees: A new classification for the genus *Cattleya* (Epidendreae; Epidendroideae; Orchidaceae). Phytotaxa.

[13] Peraza-Flores L.N., Carnevali G., Van den Berg C. (2017). A molecular phylogeny of the *Laelia* Alliance (Orchidaceae) and reassessment of *Laelia* and *Schomburgkia*. Taxon.

[14] Dalström S., Higgins W.E. (2016). New combinations and transfers to *Odontoglossum* Oncidiinae (Orchidaceae): Avoid creating new names. Harv. Pap. Bot..

[15] Yeung E.C. (2017). A perspective on orchid seed and protocorm development. Bot. Stud..

[16] Oneal E., Willis J.H., Franks R. (2010). Disruption of endosperm development is a major cause of hybrid seed inviability between *Mimulus guttatus* and *M. nudatus*. New Phytol..

[17] Teixeira da Silva J.A., Cardoso J.C., Dobránszki J., Zeng S. (2015). *Dendrobium* micropropagation: A review. Plant Cell Rep..

[18] Teixeira da Silva J.A., Tsavkelova E.A., Ng T.B., Parthibhan S., Dobránszki J., Cardoso J.C., Rao M.V., Zeng S. (2015). Asymbiotic in vitro seed propagation of *Dendrobium*. Plant Cell Rep..

[19] Li Y.-Y., Chen X.-M., Zhang Y., Cho Y.-H., Wang A.-R., Yeung E.C., Zeng X., Guo S.-X., Lee Y.-I. (2018). Immunolocalization and changes of hydroxyproline-rich glycoproteins during symbiotic germination of *Dendrobium officinale*. Front. Plant Sci..

[20] Mala B., Kuegkong K., Sa-ngiaemsri N., Nontachaiyapoom S. (2017). Effect of germination media on in vitro symbiotic seed germination of three *Dendrobium* orchids. S. Afr. J. Bot..

[21] Arditti J. (1967). Factors affecting the germination of orchid seeds. Bot. Rev..

[22] Santos S.A., Smidt E.C., Padial A.A., Ribas L.L.F. (2016). Asymbiotic seed germination and in vitro propagation of *Brasiliorchis picta*. Afr. J. Biotech..

[23] Kunakhonnuruk B., Inthima P., Kongbangkerd A. (2018). In vitro propagation of *Epipactis flava* Seidenf, an endangered rheophytic orchid: A first study on factors affecting asymbiotic seed germination, seedling development and greenhouse acclimatization. Plant Cell Tissue Organ Cult..

[24] Rao A.N., Reinert J., Bajaj Y.P.S. (1977). Tissue culture in orchid industry. Applied and Fundamental Aspects of Plant Cell Tissue and Organ Culture.

[25] Parmar G., Pant B. (2016). In vitro seed germination and seedling development of the orchid *Coelogyne stricta* (D. Don) Schltr. Afr. J. Biotechnol..

[26] Huang H., Zi X.-M., Lin H., Gao J.-Y. (2018). Host-specificity of symbiotic mycorrhizal fungi for enhancing seed germination, protocorm formation and seedling development of over-collected medicinal orchid, *Dendrobium devonianum*. J. Microb..

[27] Fochi V., Chitarra W., Kohler A., Voyron S., Singan V.R., Lindquist E.A., Barry K.W., Girlanda M., Grigoriev I.V., Martin F. (2016). Fungal and plant gene expression in the *Tulasnella calospora-Serapias vomeracea* symbiosis provides clues about nitrogen pathways in orchid mycorrizas. New Phytol..

[28] Lee Y., Hsu S., Yeung E.C. (2013). Orchid protocorm-like bodies are somatic embryos. Am. J. Bot..

[29] Fang S., Chen J.C., WEI M.J. (2016). Protocorms and protocorm-like bodies are molecularly distinct from zygotic embryonic tissues. Plant Physiol..

[30] Zanello C.A., Cardoso J.C. (2019). PLBs induction and clonal plantlet regeneration from leaf segment of commercial hybrids of *Phalaenopsis*. J. Hortic. Sci. Biotech..

[31] Chookoh N., Chiu Y., Chang C., Hu W., Dai T. (2019). Micropropagation of *Tolumnia* orchids through induction of protocorm-like bodies from leaf segments. Hortscience.

[32] Li S.H., Kuoh C.S., Chen Y.H., Chen H.H., Chen W.H. (2005). Osmotic sucrose enhancement of single-cell embryogenesis and transformation efficiency in *Oncidium*. Plant Cell Tissue Organ Cult..

[33] Naing A.H., Chung J.D., Park I.S., Lim K.B. (2011). Efficient plant regeneration of the endangered medicinal orchid, *Coelogyne cristata* using protocorm-like bodies. Acta Physiol. Plant.

[34] Kalyan K., Sil S. (2015). Protocorm-like bodies and plant regeneration from foliar explants of *Coelogyne flaccida*, a horticulturally and medicinally important endangered orchid of eastern himalaya. Lanke.

[35] Picolotto D.R.N., Paiva Neto V.B., Barros F., Padilha D.R.C., Cruz A.C.F., Otoni W.C. (2017). Micropropagation of *Cyrtopodium paludicolum* (Orchidaceae) from root tip explants. Crop Breed. App. Biotech..

[36] Samala S., Te-chato S., Yenchon S., Thammasiri K. (2014). Protocorm-like body of *Grammatophyllum speciosum* through asymbiotic seed germination. ScienceAsia.

[37] Chen C. (2016). Cost analysis of plant micropropagation of *Phalaenopsis*. Plant Cell Tissue Organ Cult..

[38] Tanaka M., Sakanishi Y. (1977). Clonal propagation of *Phalaenopsis* by leaf culture. Am. Orc. Soc. Bull..

[39] Tanaka M., Sakanishi Y., Kashemsanta M.R.S. (1980). Clonal propagation of *Phalaenopsis* through tissue culture. Proc. 9th World Orchid Conference.

[40] Tanaka M., Sakanishi Y. (1985). Regenerative capacity of in vitro cultured leaf segments excised from mature *Phalaenopsis* plants. Bull. Univ. Osaka Ser. B.

[41] Tokuhara K., Mii M. (1993). Micropropagation of *Phalaenopsis* and *Doritaenopsis* by culturing shoot tips of flower stalk buds. Plant Cell Rep..

[42] Ishii Y., Takamura T., Goi M., Tanaka M. (1998). Callus induction and somatic embryogenesis of *Phalaenopsis*. Plant Cell Rep..

[43] Vacin E.F., Went F.W. (1949). Some pH in nutrient solutions. Bot. Gaz..

[44] Huan L.V.T., Takamura T., Tanaka M. (2004). Callus formation and plant regeneration from callus through somatic embryo structures in *Cymbidium* orchid. Plant Sci..

[45] Ulisses C., Pereira J.A.F., Silva S.S., Arruda E., Morais M. (2016). Indução e histologia de embriões somáticos primários e secundários do híbrido *Phalaenopsis* Classic Spotted Pink (Orchidaceae). Acta Biol. Col..

[46] Goussard P.G., Wiid J., Kasdor G.G.F. (1991). The effectiveness of in vitro somatic embryogenesis in eliminating fanleaf virus and leafroll associated viruses from grapevines. S. Afr. J. Enol. Vitic..

[47] Quainoo A.K., Wetten A.C., Allainguillaume J. (2008). The effectiveness of somatic embryogenesis in eliminating the cocoa swollen shoot virus from infected cocoa trees. J. Virol. Met..

[48] Gambino G., Di Matteo D., Gribaudo I. (2009). Elimination of *Grapevine fanleaf virus* from three *Vitis vinifera* cultivars by somatic embryogenesis. Eur. J. Plant Pathol..

[49] Nkaa F.A., Ene-Obong E.E., Taylor N., Fauquet C., Mbanaso E.N.A. (2013). Elimination of African Cassava Mosaic Virus (ACMV) and East African Cassava Mosaic Virus (EACMV) from cassava (*Manihot esculenta* Crantz) cv. ‘Nwugo’ via somatic embryogenesis. Am. J. Biotech. Molec. Sci..

[50] Chai M.L., Xu C.-J., Senthil K., Kim J.Y. (2002). Stable transformation of protocorm-like bodies in *Phalaenopsis* orchid mediated by *Agrobacterium tumefasciens*. Sci. Hort..

[51] Mishiba K., Chin D.P., Mii M. (2005). *Agrobacterium*-mediated transformation of *Phalaenopsis* by targeting protocorms at an early stage after germination. Plant Cell Rep..

[52] Huang Y.W., Tsai Y.J., Chen F.C. (2014). Characterization and expression analysis of somatic embryogenesis receptor-like kinase genes from *Phalaenopsis*. Genet. Mol. Res..

[53] Hecht V., Vielle-Calzada J.P., Hartog M.V., Schmidt E.D., Boutilier K., Grossniklaus U., De Vries S.C. (2001). The Arabidopsis *SOMATIC EMBRYOGENESIS RECEPTOR KINASE 1* gene is expressed in developing ovules and embryos and enhances embryogenic competence in culture. Plant Physiol..

[54] Chardin C., Girin T., Roudier F., Meyer C., Krapp A. (2014). The plant RWP-RK transcription factors: Key regulators of nitrogen responses and of gametophyte development. J. Exp. Bot..

[55] Mursyanti E., Purwantoro A., Moeljopawiro S., Semiarti E. (2015). Induction of somatic embryogenesis through overexpression of ATRKD4 genes in *Phalaenopsis* “Sogo Vivien”. Ind. J. Biotech..

[56] Setiari N., Purwantoro A., Moeljopawiro S., Semiarti E. (2018). Micropropagation of *Dendrobium phalaenopsis* orchid through overexpression of embryo gene *AtRKD4*. Agriv. J. Agric. Sci..

[57] Cai J., Liu X., Vanneste K., Proost S., Tsai W.-C., Liu K.-W., Chen L.-J., He Y., Xu Q., Bian C. (2015). The genome sequence of the orchid *Phalaenopsis equestris*. Nat. Genet..

[58] Huang J.-Z., Lin C.-P., Cheng T.-C., Huang Y.-W., Tsai Y.-J., Cheng S.-Y., Chen Y.-W., Lee C.-P., Chung W.-C., Chang B.C.-H. (2016). The genome and transcriptome of *Phalaenopsis* yield insights into floral organ development and flowering regulation. PeerJ.

[59] Gow W., Chen J., Chang W. (2009). Effects of genotype, light regime, explant position, and orientation on direct somatic embryogenesis from leaf explants of *Phalaenopsis* orchid. Acta Physiol. Plant.

[60] Mehraj H., Alam M.M., Habiba S.U., Mehbub H. (2019). LEDs combined with CHO sources and CCC priming PLB regeneration of *Phalaenopsis*. Horticulture.

[61] Teixeira da Silva J.A., Winarto B. (2016). Somatic embryogenesis in two orchid genera (*Cymbidium, Dendrobium*). Meth. Mol. Biol..

[62] Reuter E. (1983). The importance of propagating *Phalaenopsis* by tissue culture. Orchid Rev..

[63] Meilasari D., Prayogo I. (2016). Regeneration of plantlets through PLB (protocorm-like body) formation in *Phalaenopsis* ‘Join Angle × Sogo Musadian’. J. Math. Fund. Sci..

[64] Chen Y.-C., Chang C., Chang W.C. (2000). A reliable protocol for plant regeneration from callus culture of *Phalaenopsis*. Vitr. Cell. Dev. Biol. Plant.

[65] Park S.Y., Murthy H.N., Paek K.Y. (2000). Mass multiplication of protocorm-like bodies using bioreactor system and subsequent plant regeneration in *Phalaenopsis*. Plant Cell Tissue Organ Cult..

[66] Tokuhara K., Mii M. (2001). Induction of embryogenic callus and cell suspension culture from shoot tips excised from flower stalk buds in *Phalaenopsis* (Orchidaceae). Vitr. Cell. Dev. Biol. Plant.

[67] Park S.-Y., Murthy H.N., Paek K.Y. (2002). Rapid propagation of *Phalaenopsis* from flower stalk-derived leaves. Vitr. Cell. Dev. Biol. Plant.

[68] Park S.Y., Yeung E.C., Chakrabarty D., Paek K.Y. (2002). An efficient direct induction of protocorm-like bodies from leaf subepidermal cells of *Doritaenopsis* hybrid using thin-section culture. Plant Cell Rep..

[69] Park S.-Y., Hosakatte N.M., Paek K.Y. (2003). Protocorm-like body induction and subsequent plant regeneration from root tip cultures of *Doritaenopsis*. Plant Sci..

[70] Tokuhara K., Mii M. (2003). Highly-efficient somatic embryogenesis from cell suspension cultures of *Phalaenopsis* orchids by adjusting carbohydrate sources. Vitr. Cell. Dev. Biol. Plant.

[71] Kuo H., Chen J., Chang W. (2005). Efficient plant regeneration through direct somatic embryogenesis from leaf explants of Phalaenopsis ‘Little Steve’. Vitr. Cell. Dev. Biol. Plant.

[72] Chen J.T., Chang W.C. (2006). Direct somatic embryogenesis and plant regeneration from leaf explants of *Phalaenopsis amabilis*. Biol. Plant.

[73] Murdad R., Hwa K.S., Seng C.K., Latip M.A., Aziz Z.A., Ripin R. (2006). High frequency multiplication of *Phalaenopsis gigantea* using trimmed bases protocorms technique. Sci. Hortic..

[74] Minamiguchi J., Machado Neto N.B. (2007). Embriogênese somática direta em folhas de *Phalaenopsis*: Orchidaceae. Colloq. Agrar..

[75] Sinha P., Hakim M., Alam M. (2007). Efficient micropropagation of *Phalaenopsis amabilis* (L.) BL. cv. ’Cool Breeze’ using inflorescence axis thin sections as explants. Propag. Ornam. Plants.

[76] Ling A.C.K., Yap C.P., Shaib J.M., Vilasini P. (2007). Induction and morphogenesis of *Phalaenopsis* callus. J. Trop. Agric. Food Sci..

[77] Gow W., Chen J., Chang W. (2008). Influence of growth regulators on direct embryo formation from leaf explants of *Phalaenopsis* orchid. Acta Physiol. Plant..

[78] Gow W., Chen J., Chang W. (2010). Enhancement of direct somatic embryogenesis and plantlet growth from leaf explants of *Phalaenopsis* by adjusting culture period and explant length. Acta Physiol. Plant..

[79] Chen W.H., Tang C.Y., Kao Y.L. (2009). Ploidy doubling by in vitro culture of excised protocorms or protocorm-like bodies in *Phalaenopsis* species. Plant Cell Tissue Organ Cult..

[80] Subramaniam S., Balasubramaniam V.R.M.T., Poobathy R., Sasidharan S. (2009). Chemotaxis Movement and Attachment of *Agrobacterium tumefaciens* to *Phalaenopsis violacea* Orchid Tissues an Assessment of Early Factors Influencing the Efficiency of Gene Transfer. Trop. Life Sci. Res..

[81] Sinha P., Jahan M.A.A., Munshi J.L., Khatun R. (2010). High frequency regeneration of *Phalaenopsis amabilis* (L.) Bl. cv. Lovely through in vitro culture. Plant Tissue Cult. Biotech..

[82] Khoddamzadeh A.A., Sinniah U.R., Kadir M.A., Kadzimin S.B., Mahmood M., Sreeramanan S. (2010). Detection of somaclonal variation by random amplified polymorphic DNA analysis during micropropagation of *Phalaenopsis bellina* (Rchb.f.) Christenson. Afr. J. Biotech..

[83] Khoddamzadeh A.A., Sinniah U.R., Kadir M.A., Kadzimin S.B., Mahmood M., Sreeramanan S. (2011). In vitro induction and proliferation of protocorm-like bodies (PLBs) from leaf segments of *Phalaenopsis bellina* (Rchb.f.) Christenson. Plant Grow. Reg..

[84] Niknejad A., Kadir M.A., Kadzimin S.B. (2001). In vitro plant regeneration from protocorms-like bodies (PLBs) and callus of *Phalaenopsis gigantea* (Epidendroideae: Orchidaceae). Afr. J. Biotech..

[85] Sinha P., Jahan M.A.A. (2011). Clonal propagation of *Phalaenopsis amabilis* (L.) BL. Cv. ‘Golden Horizon’ through in vitro culture of leaf segments. BangladeshJ. Sci. Ind. Res..

[86] Van Thanh P., Teixeira Da Silva J.A., Huy H.E., Tanaka M. (2011). The effects of permanent magnetic fields on in vitro growth of *Phalaenopsis* plantlets. J. Hortic. Sci. Biotech..

[87] Rittirat S., Kongruk S., Te-Chato S. (2012). Induction of protocorm-like bodies (PLBs) and plantlet regeneration from wounded protocorms of *Phalaenopsis cornu-cervi* (Breda) Blume & Rchb. f. J. Agric. Tech..

[88] Samarfard S., Kadir M.A., Kadzimin S.B., Ravanfar S., Saud H.M. (2013). Genetic stability of in vitro multiplied *Phalaenopsis gigantea* protocorm-like bodies as affected by chitosan. Not. Bot. Horti Agrobot..

[89] Antensari F., Mariani T.S., Wicaksono A. (2014). Micropropagation of *Phalaenopsis* ‘R11 × R10’ Through Somatic Embryogenesis Method. Asian J. Appl. Sci..

[90] Huang Y.-W., Tsai Y.-J., Cheng T.-C., Chen J.-J., Chen F.C. (2014). Physical wounding and ethylene stimulated embryogenic stem cell proliferation and plantlet regeneration in protocorm-like bodies of *Phalaenopsis* orchids. Genet. Mol. Res..

[91] Rittirat S., Klaocheed S., Thammasiri K. (2014). Enhanced efficiency for propagation of *Phalaenopsis cornu-cervi* (Breda) Blume & Rachb. F. using trimmed leaf technique. Int. J. Agric. Biosyst. Eng..

[92] Samarfard S., Kadir M.A., Kadzimin S.B., Saud H.M., Ravanfar S.A., Danaee M. (2014). In vitro propagation and detection of somaclonal variation in *Phalaenopsis gigantea* as affected by chitosan and thidiazuron combinations. Hortscience.

[93] Soe K.W., Myint K.T., Naing A.H., Kim C.K. (2014). Optimization of efficient protocorm-like body (PLB) formation of *Phalaenopsis* and *Dendrobium* hybrids. Curr. Res. Agric. Life Sci..

[94] Feng J., Chen J. (2014). A novel in vitro protocol for inducing direct somatic embryogenesis in *Phalaenopsis aphrodite* without taking explants. Sci. World J..

[95] Balilashaki K., Vahedi M., Karimi R. (2015). In vitro direct regeneration from node and leaf explants of *Phalaenopsis* cv. ‘Surabaya’. Plant Tissue Cult. Biotech..

[96] Sultana K.S., Hasan K.M., Hasan K.M., Sultana S., Mehraj H., Ahasan M., Shimasaki K., Habiba S.U. (2015). Effect of two elicitors on organogenesis in protocorm-like bodies (PLBs) of *Phalaenopsis* ‘Fmk02010’ cultured *in vitro*. World Appl. Sci. J..

[97] Balilashaki K., Ghehsareh M.G. (2016). Micropropagation of *Phalaenopsis amabilis* var. ’Manila’ by leaves obtained from in vitro culturing the nodes of flower stalks. Not. Sci. Biol..

[98] Mose W., Indrianto A., Purwantoro A., Semiarti E. (2017). The influence of Thidiazuron on direct somatic embryo formation from various types of explant in *Phalaenopsis amabilis* Blume Orchid. Hayati J. Biosci..

[99] Murashige T., Skoog F. (1962). A revised medium for rapid growth and bio assays with tobacco tissue cultures. Physiol. Plant..

[100] Kano K. (1965). Studies on the media for orchid seed germination. Mem. Fac. Agri. Kagawa Univ..

[101] Ernst R. (1994). Effects of thidiazuron on in vitro propagation of *Phalaenopsis* and *Doritaenopsis* (Orchidaceae). Plant Cell Tissue Organ Cult..

[102] Semiarti E., Indrianto A., Purwantoro Y.H., Martiwi I.N.A., Feroniasanti Y.M.A., Nadifah F., Mercuriana I.S., Dwiyani R., Iwakawa H., Yoshioka Y. (2010). High-frequency genetic transformation of *Phalaenopsis amabilis* orchid using tomato extract-enriched medium for the pre-culture of protocorms. J. Hortic. Sci. Biotech..

[103] Chuanjun X., Zhiwei R., Ling L., Biyu Z., Junmei H., Wen H., Ou H. (2015). The effects of polyphenol oxidase and cycloheximide on the early stage of Browning in *Phalaenopsis* explants. Hortic. Plant J..

[104] Novak S.D., Luna L.J., Gamage R.N. (2014). Role of auxin in orchid development. Plant Sign. Behav..

[105] Bairu M.W., Aremu A.O., Van Staden J. (2011). Somaclonal variation in plants: Causes and detection methods. Plant Grow. Reg..

[106] Raynalta E., Elina J., Sudarsono S., Sukma D. (2018). Clonal fidelity of micropropagated *Phalaenopsis* plantlets based on assessment using eighteen Ph-Pto SNAP marker loci. J. Agric. Sci..

[107] Park S.I., Yeung E.C., Paek K.Y. (2010). Endoreduplication in *Phalaenopsis* is affected by light quality from light-emitting diodes during somatic embryogenesis. Plant Biotec. Rep..

[108] Young P.S., Murthy H.N. (2000). Clonal fidelity of micropropagated *Phalaenopsis* plantlets based on assessment using eighteen Ph-Pto SNAP marker loci. Yoeup, P.K. Mass multiplication of protocorm-like bodies using bioreactor system Clonal fidelity of micropropagated *Phalaenopsis* plantlets based on assessment using eighteen Ph-Pto SNAP marker loci. and subsequent plant regeneration in *Phalaenopsis*. Plant Cell Tissue Organ Cult..

[109] Liu M., Xu Z., Yang Y., Feng Y. (2011). Effects of different spectral lights on *Oncidium* PLBs induction, proliferation, and plant regeneration. Plant Cell Tissue Organ Cult..

[110] Wei C.H. (2007). Optimization of PLB induction conditions for *Oncidium*. Fuj. J. Agr. Sci..

[111] Li W.-L., Zhai L.-S., Ziu Y.-P. (2004). Study on induction and culture of *Oncidium* protocorm-like body (PLB). Hen. Sci..

[112] Tran M.V., Nguyen K.V., Hoa B.T. Rapid micropropagation of Vu Nu Orchid (*Oncidium* sp.) by using tissue culture technique. Proceedings of the CBU International Conference.

[113] Yang J.F., Piao X.C., Sun D., Lian M.L. (2010). Production of protocorm-like bodies with bioreactor and regeneration in vitro of *Oncidium* ‘Sugar Sweet’. Sci. Hortic..

[114] Chen J.T., Chang W.C. (2001). Effects of auxins and cytokinins on direct somatic embryogenesis from leaf explants of *Oncidium* ‘Gower Ramsey’. Plant Growth Regul..

[115] Chen J.-T., Chang C., Chang W.C. (1999). Direct somatic embryogenesis from leaf explants of *Oncidium* ‘Gower Ramsey’ and subsequent plant regeneration. Plant Cell Rep..

[116] Chen J.-T., Chang W.C. (2000). Plant regeneration via embryo and shoot bud formation from flower-stalk explants of *Oncidium* Sweet Sugar. Plant Cell. Tiss. Organ Cult..

[117] Chen J., Chang W. (2000). Efficient plant regeneration through somatic embryogenesis from callus cultures of *Oncidium* (Orchidaceae). Plant Sci..

[118] Chen J.-T., Chang W.-C. (2003). Effects of GA_3_, ancymidol, cycocel and paclobutrazol on direct somatic embryogenesis of *Oncidium* in vitro. Plant Cell Tissue Organ Cult..

[119] Kerbauy G.B. (1984). Plant regeneration of *Oncidium varicosum* (Orchidaceae) by means of root tip culture. Plant Cell Rep..

[120] Flachsland E.A., Graciela-Mroginski L.A. (2001). Regeneración de Protocormos y Yemas de *Oncidium bifolium* Sims. Por cultivo in vitro de láminas foliares. researchgate.net/publication/267300737_Regeneracion_de_protocormos_y_yemas_de_Oncidium_bifolium_Sims_por_cultivo_in_vitro_de_laminas_foliares.

[121] Chen J.T., Chang W.C. (2002). Effects of tissue culture conditions and explant characteristics on direct somatic embryogenesis in *Oncidium* ‘Gower Ramsey’. Plant Cell Tissue Organ Cult..

[122] Rahman S.M.M., Islam M.S., Sen P.K., Begum F. (2005). In vitro propagation of *Oncidium taka*. Biotechnology.

[123] Jheng F.Y., Do Y.Y., Liauh Y.W., Chung J.P., Huang P.L. (2006). Enhancement of growth and regeneration efficiency from embryogenic callus cutures of *Oncidium* “Gower Ramsey” by adjusting carbohydrate sources. Plant Sci..

[124] Hong P.I., Chen J.T., Chang W.C. (2008). Promotion of direct somatic embryogenesis of *Oncidium* by adjusting carbon sources. Biol. Plant..

[125] Wang A.-S., Lin M.-G., Liu F.-X. (2009). Rapid propagation of cut flower varieties of *Oncidium* by tissue culture. Guangxi Agric. Sci..

[126] Chung J.P., Huang C.Y., Dai T.E. (2010). Spectral effects on embryogenesis and plantlet growth of *Oncidium* Gower Ramsey. Sci. Hortic..

[127] Mayer J.L.S., Stancato G.C., Appezzato-da-Glória B. (2010). Direct regeneration of Protocorm-like bodies (PLBs) from leaf apices of *Oncidium flexuosum* Sims (Orchidaceae). Plant Cell Tissue Organ Cult..

[128] Chen J.-T. (2012). Induction of petal-bearing embryos from root-derived callus of *Oncidium* ‘Gower Ramsey’. Acta Physiol. Plant.

[129] Gomes L.R.P., Franceschi C.R.B., Ribas L.L.F. (2015). Micropropagation of *Brasilidium forbesii* (Orchidaceae) through transverse and longitudinal thin cell layer culture. Acta Sci. Biol. Sci..

[130] Mahesh R., Kumar H.G.A., Satyanarayana S. (2018). Synthesis and characterization of 2-mercapto-N methyl imidazole substituted benzimidazole derivatives and investigation of their effect on production of plantlets in *Oncidium* Gower Ramsey. Mater. Today Proc..

[131] Lloyd G., McCown B. (1980). Commercially-feasible micropropagation of mountain laurel, *Kalmia latifolia*, by use of shoot-tip culture. Int. Plant Propag. Soc. Proc..

[132] Chen Y.H., Chang Y.S., Chen W.H. (2001). Tissue culture advances for mass propagation of *Oncidium* mericlones. Rep. Taiwan Sugar Res. Inst..

[133] Wang T.C., Zhang M., Tong Y.O. (2019). Molecular specstrum of somaclonal variation in PLB-regenerated *Oncidium* revealed by SLAF-seq. Plant Cell Tissue Organ Cult..

[134] Mohanty P., Paul S., Das M.C., Kumaria S., Tandon P. (2012). A simple and efficient protocol for the mass propagation of *Cymbidium mastersii*: An ornamental orchid from Northeast India. Aob Plants..

[135] Teixeira da Silva J.A., Tanaka M. (2006). Multiple regeneration pathways via Thin Cell Layers in hybrid *Cymbidium* (Orchidaceae). J. Plant Growth Regul..

[136] Malabadi R.B., Mulgund G.S., Kallappa N. (2005). Micropropagation of *Dendrobium nobile* from shoot tip sections. J. Plant Physiol..

[137] Parthibhan S., Venkateswara Rao M., Teixeira da Silva J.A., Senthil Kumar T. (2018). Somatic embryogenesis from stem thin cell layers of *Dendrobium aqueum*. Biol. Plant..

[138] Teixeira da Silva J.A., Jin X., Dobránszki J., Lu J., Wang H., Zotz G., Cardoso J.C., Zeng S. (2016). Advances in *Dendrobium* molecular research: Applications in genetic variations, identification and breeding. Mol. Phylogen. Evol..

[139] Bhattacharyya P., Kumaria S., Tandon P. (2016). High frequency regeneration protocol for *Dendrobium nobile*: A model tissue culture approach for propagation of medicinally important orchid species. S. Afr. J. Bot..

[140] Chien K.W., Agrawal D.C., Tsay H.S., Chang C.A. (2015). Elimination of mixed ‘*Odontoglossum ringspot*’ and ‘*Cymbidium mosaic*’ *viruses* from *Phalaenopsis* hybrid ‘V3’ through shoot-tip culture and protocorm-like body selection. Crop Protect..

[141] Chen F.C. (2009). Phalaenopsis in Vitro Cloning: Strategy for PLB or Shoots?.

[142] Miguel T.P., Leonhardt K.W. (2011). In vitro polyploid induction of orchids using oryzalin. Sci. Hortic..

[143] Sarathum S., Hegele M., Tantiviwat S., Nanakorn M. (2010). Effect of concentration and duration of colchicine treatment on polyploid induction in *Dendrobium scabrilingue* L.. Eur. J. Hort. Sci..

[144] Wannajindaporn A., Kativat C., Tantasawat P.A. (2016). Mutation induction of *Dendrobium* ‘Earsakul’ using sodium azide. Hortscience.

[145] Billore V., Mirajkar S.J., Suprasanna P., Jain M. (2019). Gamma irradiation induced effects on in vitro shoot cultures and influence of monochromatic light regimes on irradiated shoot cultures of *Dendrobium sonia* orchid. Biotech. Rep..

[146] Chew Y.-C., Abdullah W., Kok D.A., Ong-Abdullah J., Mahmood M., Lai K.-S. (2019). Development of an efficient particle bombardment transformation system for de endemic orchid *Phalaenopsis bellina*. Sains Malays..

[147] Mii M., Chin D.P., Lee Y.I., Yeung E.T. (2018). Genetic transformation of orchid species: An overview of approaches and methodologies. Orchid Propagation: From Laboratories to Greenhouses – Methods and Protocol.

[148] Liau C.-H., You S.-J., Prasad V., Hsiao H.-H., Lu J.-C., Yang N.-S., Chan M.-T. (2003). *Agrobacterium tumefasciens*-mediated transformation of *Oncidium* orchid. Plant Cell Rep..

[149] Thiruvengadam M., Hsu W.-H., Yang C.-H. (2011). Phosphomannose-isomerase as a selectable marker to recover transgenic orchid plants (*Oncidium* Gower Ramsey). Plant Cell Tissue Organ Cult..

[150] Morel G.M. (1960). Producing virus free Cymbidiums. Am. Orchid Soc. Bull..

[151] Pradhan S., Regmi T., Ranjit M., Pant B. (2016). Production of virus-free orchid *Cymbidium aloifolium* (L.) Sw. by various tissue culture techniques. Heliyon.

[152] Khentry Y., Paradornuwat A., Tantiwiwat S., Phansiri S., Thaveechail N. (2006). Incidence of *Cymbidium mosaic virus* and *Odontoglossum Ringspot Virus* on in vitro Thai native orchid seedlings and cultivated orchid Mericlones. Kasertsat J. Nat. Sci..

[153] Shen R.-S., Hsu S.-T., Lee Y.I., Yeung E.T. (2018). Virus elimination through meristem culture and rapid clonal propagation using a temporary immersion system. Orchid Propagation: From Laboratories to Greenhouses–Methods and Protocols.

[154] Saiprasad G.V.S., Polisetty R. (2003). Propagation of three orchid genera using encapsulated protocorm-like bodies. Vitr. Cell. Dev. Biol. Plant.

[155] Wang H.-Q., Jin M.-Y., Paek K.-Y., Piao X.-C., Lian M.-L. (2016). An efficient strategy for enhancement of bioactive compounds by protocorm-like body culture of *Dendrobium candidum*. Ind. Crop. Prod..

[156] Paek K.Y., Hahn E.J., Park S.Y. (2011). Micropropagation of *Phalaenopsis* orchids via protocorms and protocorm-like bodies. Methods Mol. Biol..

[157] Hsu C.-C., Lai P.-H., Chen T.-C., Tsai W.-C., Hsu J.-L., Hsiao Y.-Y., Wu W.-L., Tsai C.-H., Chen W.-H., Chen H.-H. (2019). PePIF1, a P-lineage of PIF-like transposable element identified in protocorm-like bodies of Phalaenopsis orchids. Bmc Genom..

[158] Chin C.K., Lee Z.H., Mubbarakh S.A., Antony J.J.J., Chew B.L., Subramanian S. (2019). Effects of plant growth regulators and activated charcoal on somaclonal variations of protocorm-like bodies (PLBs) of *Dendrobium* Sabin Blue orchid. Biocatal. Agric. Biotech..

[159] Chen W.H., Chen T.M., Fu Y.M., Hsieh R.M. (1998). Studies on somaclonal variation in *Phalaenopsis*. Plant Cell Rep..

[160] Bairu M.W., Stirk W.A., Dolezal K., van Staden J. (2008). The role of topolins in micropropagation and somaclonal variation of banana cultivars ‘Williams’ and ‘Grand Naine’ (*Musa* spp. AAA). Plant Cell Tissue Organ Cult..

[161] Tokuhara K., Mii M. (1998). Somaclonal variation in flower and inflorescence axis in micropropagated plants through flower stalk bud culture of *Phalaenopsis* and *Doritaenopsis*. Plant Biotech..

[162] Chen Y.H., Tsai Y.J., Huang J.Z., Chen F.C. (2005). Transcription analysis of peloric mutants of *Phalaenopsis* orchids derived from tissue culture. Cell Res..

[163] Antony J.J.J., Shanshir R.A., Poobathy R., Chew B.L., Subramanian S. (2015). Somaclonal variations were not induced by cryopreservation: Levels of somaclonal variation of in vitro and thawed protocorms of *Dendrobium* Bobby Messina analysed by SCoT and TRAP DNA markers. South Afr. J. Bot..

[164] Chen F.-C., Yu J.-Y., Chen P.-Y., Huang Y.-W. (2008). Somaclonal variation in orchids and the application of biotechnology. Acta Hortic..

